# Rational drug design of CB2 receptor ligands: from 2012 to 2021

**DOI:** 10.1039/d2ra05661e

**Published:** 2022-12-08

**Authors:** Yan-ran Wu, Jia-qin Tang, Wan-nian Zhang, Chun-lin Zhuang, Ying Shi

**Affiliations:** School of Pharmacy, Key Laboratory of Hui Ethnic Medicine Modernization, Ministry of Education, Ningxia Medical University 1160 Shengli Street Yinchuan 750004 China nxshiying@163.com; School of Pharmacy, Second Military Medical University 325 Guohe Road Shanghai 200433 China

## Abstract

Cannabinoid receptors belong to the large family of G-protein-coupled receptors, which can be divided into two receptor types, cannabinoid receptor type-1 (CB1) and cannabinoid receptor type-2 (CB2). Marinol, Cesamet and Sativex are marketed CB1 drugs which are still in use and work well, but the central nervous system side effects caused by activation CB1, which limited the development of CB1 ligands. So far, no selective CB2 ligand has been approved for marketing, but lots of its ligands in the clinical stage and pre-clinical stage have positive effects on the treatment of some disease models and have great potential for development. Most selective CB2 agonists are designed and synthesized based on non-selective CB2 agonists through the classical med-chem strategies, *e.g.* molecular hybridization, scaffold hopping, bioisosterism, *etc.* During these processes, the balance between selectivity, activity, and pharmacokinetic properties needs to be achieved. Hence, we summarized some reported ligands on the basis of the optimization strategies in recent 10 years, and the limitations and future directions.

## Abstract 2

Cannabinoid receptors belong to the large family of G-protein-coupled receptors, which can be divided into two receptor types, cannabinoid receptor type-1 (CB1) and cannabinoid receptor type-2 (CB2).

## Graphical abstract

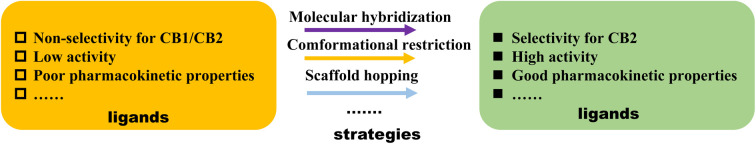

## 1. Introduction

Cannabinoid Receptors (CB) belonged to g-protein-coupled Receptors of type A, including at least two receptor types, namely, cannabinoid receptor type-1 (CB1) and cannabinoid receptor type-2 (CB2).^1,2^ The protein crystals of both CB1 and CB2 had been resolved,^3,4^ which could not only shorten drug development cycles but also enhanced our understanding of the ligand induced inactive or active-like states. The CB1 was composed of 472 amino acids and mainly distributed in the brain, especially in the hippocampus, cortex, basal ganglia and cerebellum, but it was also present in numerous peripheral tissues.^5–7^ CB2 was composed of 360 amino acids and was located primarily in peripheral nerve tissues: immune organs hijocytes, spleen and adrenal glands, heart, lungs, prostate, uterus, pancreas and testes, it was also present in the CNS.^8,9^ The two cannabinoid receptors shared 44% homology and 68% sequence similarity in the transmembrane regions.^10^ Activation of CB1 regulated energy metabolism disorders such as appetite regulation, obesity, and anorexia.^11^ Rimonabant was approved in Europe in 2006 as a reverse agonist or antagonist of the CB1 for the treatment of obesity.^12^ However, it was withdrawn two years later due to central nervous system side effects such as depression, anxiety and suicidal thoughts.^13^ Although rimonabant was withdrawn due to side effects, other marketed CB1 drugs such as Marinol, Cesamet and Sativex are still in use and work well. Studies have shown that activating CB2 can also cause side effects such as immunosuppression. So far, no selective CB2 ligand has been approved for marketing, but lots of its ligands in the clinical stage and pre-clinical stage have positive effects on the treatment of some disease models and have great potential for development. CB2 was associated with a variety of diseases in humans, ranging from cardiovascular, gastrointestinal, liver, kidney, neurodegenerative, psychiatric, bone, skin, autoimmune, lung disorders to pain and cancer.^14^ All these diseases could be regulated *via* CB2, which based on some typically cellular signalling including the activation of mitogen-activated protein kinases and JUN N terminal kinases, as well as a transient increase in the intracellular calcium levels, resulting in complex physiological functions.^15^

Most of the CB2 ligands possessed poor pharmacokinetic properties including high lipophilicity, low solubility, tight plasma protein binding, high *in vivo* clearance and low oral bioavailability.^16^ Clearly, a balance needed to be established between excellent pharmacokinetic properties and high activity and selectivity for CB2. Synthetic cannabinoids may be expected to achieve this goal through rational drug design and structural optimization strategies. The first selective ligands designed and synthesized by Huffman's group, in particular JWH-133 and other analogs.^17^ This also happens with the selective agonists designed and synthesized by Jagerovic's group using chromenopyrazole or isoxazole structures (*e.g.* PM-226).^18^ Competition binding experiments were performed by using [^3^H]CP-55,940, and potency experiments contained TRPV1 and TRPA1 channel assays,^19^ cAMP accumulation assay, the [^35^S]GTPγS assays^20^ and calcium mobilization assay.^21^

The mechanisms of action of CB1 and CB2 were revealed *via* the antagonist- and agonist-bound crystal structures.^22^ When CB1 was activated, the conformational changes appeared in both extracellular and intracellular. On the contrary, CB2 was activated *via* agonists and accompanied conformational changes only in the intracellular part. These two receptors achieved the conformational changes and trigger receptor activation and downstream signal transduction based on the toggle switch residues, CB1 dependent on Phe3.36 and Trp6.48, yet CB2 rely on Trp6.48. Additionally, there were some differences in receptor–Gi interaction surface between CB1 and CB2. For example, the intracellular part of helix V in CB1 extends during activation, while this region in CB2 not only extended, but also shifted outward by about 6 Å due to the extra bending flexibility offered by residue Gly5.59 (Met5.59 in CB1). In contrast, the unique residue P139ICL2 in CB2 was away from the hydrophobic interaction network that probably limited CB2 to only specifically couple with Gi. In agreement, mutation of P139ICL2 into Phe or Leu enabled CB2 to couple with Gs.^23^ Here we summarized some drug design and structural optimization strategies which were used to improve activity and selectivity for CB2 or optimize pharmacokinetic properties.

## 2. Strategies of optimization

### 2.1 Molecular hybridization

Xie's group designed and synthesized compound 2, which was a novel chemotype with a trisubstituted sulfonamide scaffold and the binding activity of CB2 achieved to 750 nM. Compared with the structure of 1 and considering the QSAR results, they concluded that a longer chain in blue zone was considerable important for the CB2 inverse agonist. After recombination the pivotal group, Compound 3 was generated with a diethylamino group and confirmed to have a much better binding affinity of CB2. After further optimization, the trisubstituted sulfonamide compound 4 was generated, which exhibited the potent selective CB2 inverse agonism and great inhibition of osteoclast formation.^24^
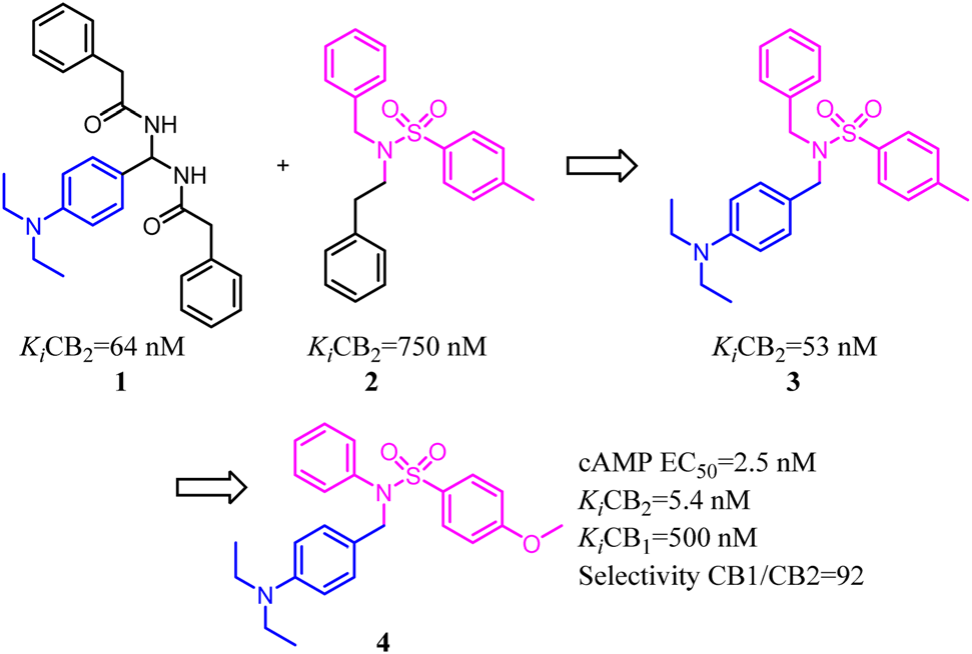


A set of 2-pyridone analogs and 2-quinolone derivatives belonged to the selective CB2 ligands which were selected to build up the CoMFA model to elucidate the essentially structural requirements responsible for the binding of ligands to the CB2.^25^7 was designed by combining the privileged groups of 2-pyridone analogs and 2-quinolone derivatives, which were based on the CoMFA model. These compounds were evaluated by calcium mobilization assays as the highly selective ligands for the CB2.
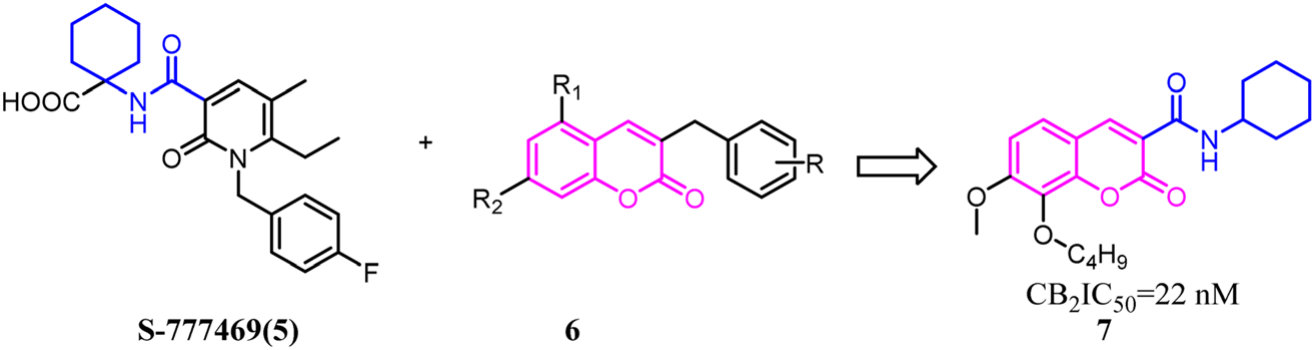


Baraldi's group designed novel oxazinoquinoline CB2 agonists by chemical structure hybridization that incorporated the privileged structures of quinolone compound 8 and the cannabimimetic indole 9, which possessed potent cannabinoid agonistsm with high selectivity for the CB2 *versus* CB1 receptor. Further identification and optimization led to a series of 7-oxo-[1,4]oxazino[2,3,4-*ij*]quinoline-6-carboxamide derivatives which contained 10 with a elevation of both binding activity and agonism.^26^
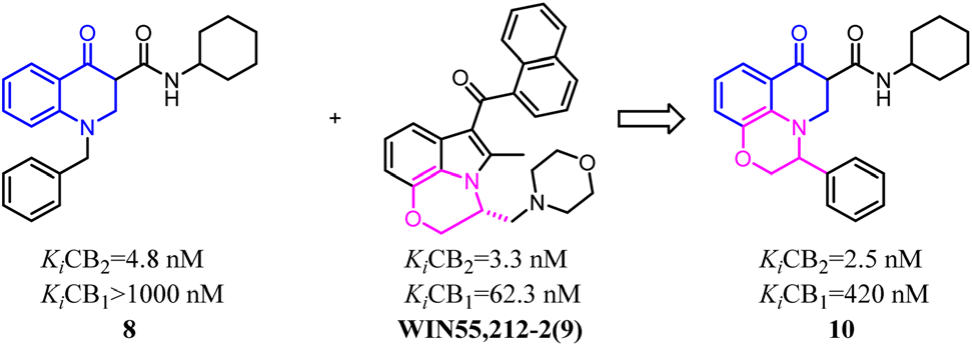


HU-308 was a full agonist of CB2 with high affinity (*K*_i_ hCB2 = 20 nM) and selectivity. And meanwhile, it was a pinene derivative lacking the central pyran, and possessed two methyl ethers that were the important factor to high CB2 selectivity.^27^AM841, a full agonist of both CB2 and CB1, possessed a phenol along with a primary aliphatic alcohol. Its most notable feature was the electrophilic isothiocyanate at the terminus of the aliphatic sidechain, which could enable cross-linking of protein targets.^28^ Carreira's team hybrided the privileged groups of HU-308 and AM841 to give compound 13 that emerged as a potent CB2 agonist with high selectivity.^29^
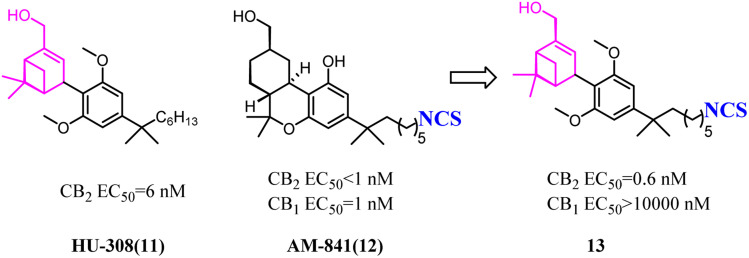


Both β-sulfonylacetamide 14 and the diazepane 15 exhibited potent CB2 agonism and high selectivity. Hickey's group combined the blue zones of 14 and pink zone of 15 to afford compound 16, and the results showed that the selectivity was improved, but the solubility was reduced.^30^ Hence keeping balance between potency and selectivity with acceptable drug-like properties including metabolic stability and permeability needed to be considered cautiously.
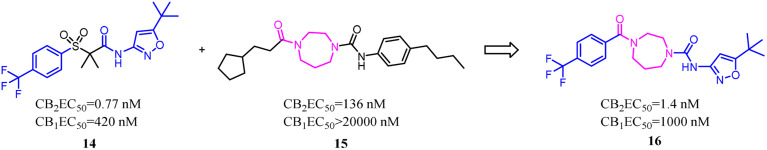


Mugnaini's team previously reported 4-hydroxy-2-quinolone-3-carboxamides based on the 4-quinolone structure, which the affinity was improved but the physicochemical properties. With the aim of maintaining the balance between hydrophilic and lipophilic for CB2 ligands, they used the molecular hybridization strategy to combine the 4-hydroxy-2-quinolone and pyrazolo[3,4-*b*]pyridine-6-one, and finally generated the new series of isosteric 7-hydroxy-pyrazolo[4,3-*b*]pyridin-5-one derivatives. These new compounds exhibited high affinity and moderate to good selectivity with good physicochemical characteristics for CB2. Among them, compound 17 emerged as a potent CB2 agonist which could reduce pain in rats carrying osteoarthritis induced by injection of monoiodoacetic acid.^31^
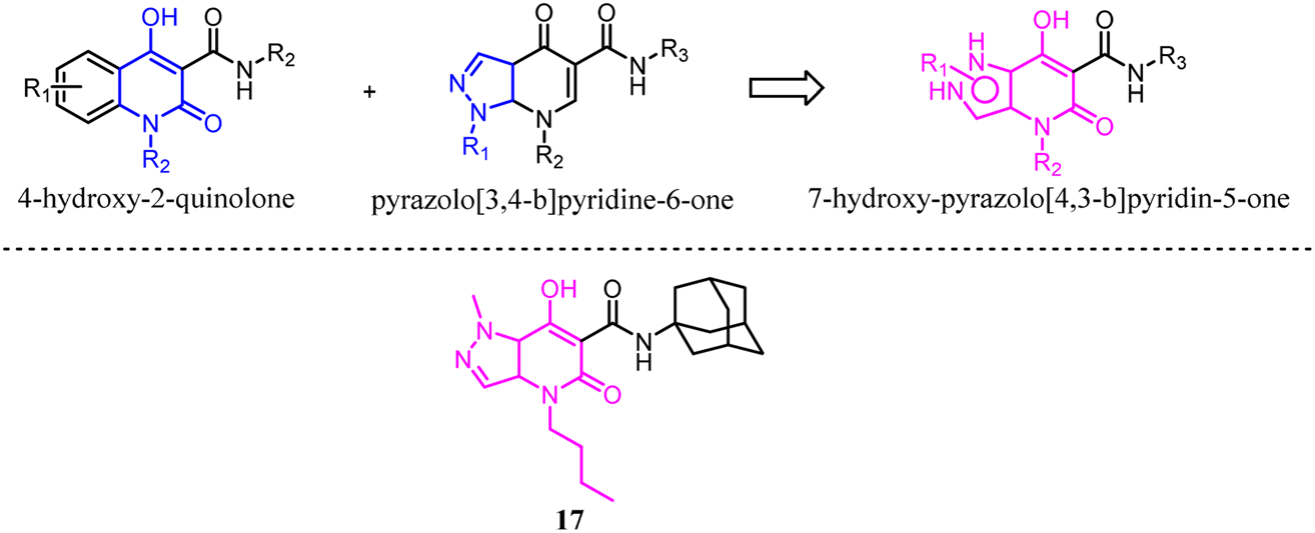


Millet's team developed a series of novel selective CB2 agonists based on a benzo[*d*]thiazol-2(3*H*)-one scaffold. They found that a long aliphatic chain extending toward a hydrophobic region of the receptor was essential for improving activity and selectivity.^32^ This drug design project led to the discovery of compound 20, a potent CB2 agonist with high selectivity. This compound exhibited no cytotoxicity, acceptable ADMET parameters and ability to counteract colon inflammatory process *in vivo*.
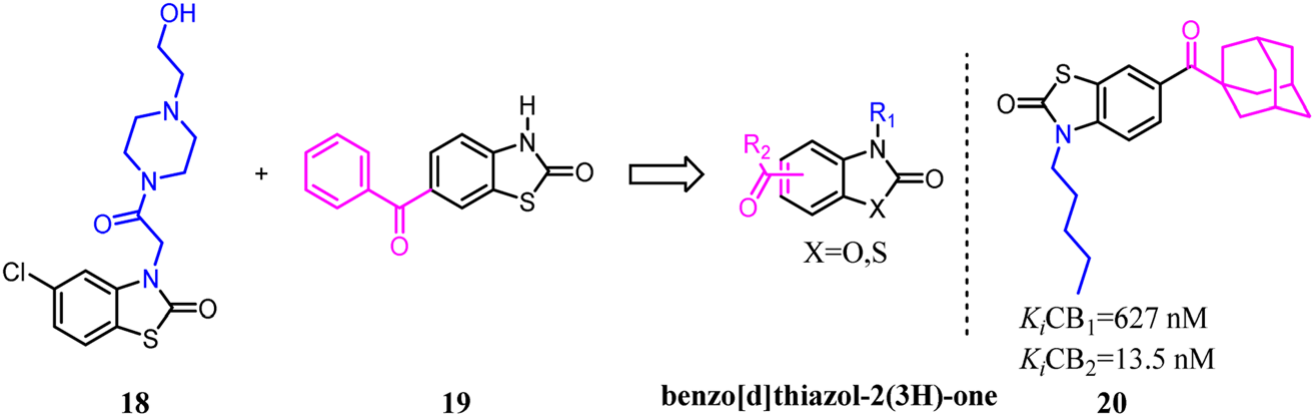


### 2.2 Scaffold hopping

The scaffold hopping strategy was a powerful approach for the discovery and modulation of medicinal ingredient by modifying the core structure of promising ligands.^29^ Furthermore, this strategy not only gave the opportunity to modulate both selectivity and affinity of a given ligand but also allowed the development and the exploitation of innovative chemistry.

1,8-Naphthyridine-4(1*H*)one-3-carboxamide derivatives were the CB2 ligands with high affinity and selectivity. To further explore structural diversity of the derivatives, the six member ring A of 1,8-naphthyridine was curtailed to five member ring imidazole, which the 1,8-naphthyridine-4(1*H*)one-3-carboxamide scaffold switched to pyrazolo[5,4-*b*]pyridin-4-one scaffold.^33^ The results showed that the most obtained compounds exhibited more significant affinity and selectivity for CB2, particularly for 21. Moreover, they found that the functionality of these ligands was controlled by the nature of the heteroaryl function condensed with the pyridine ring. In cAMP assays, the novel ligands showed dose-dependent effects on the modulation of forskolin-induced cAMP production, revealing different behaviors as full agonists, partial agonists, and inverse agonists.
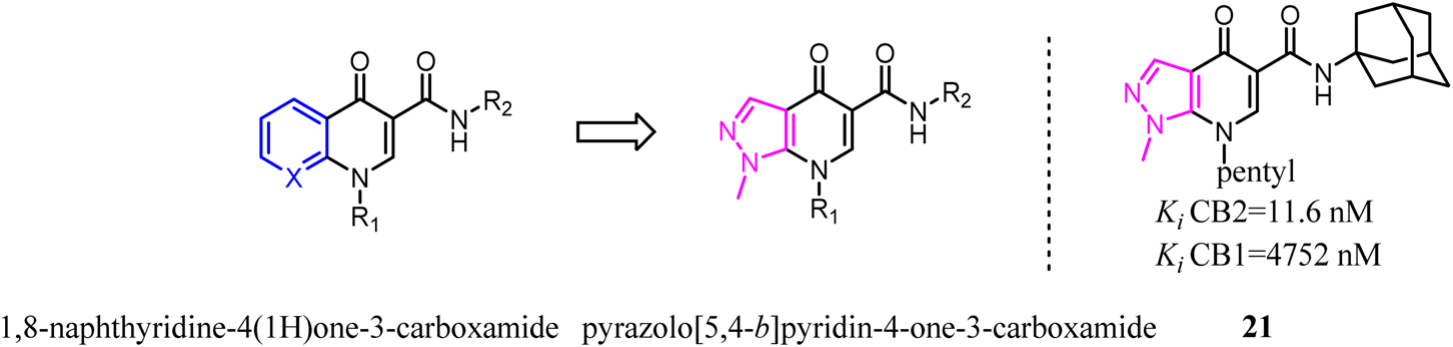


Osman's team designed and synthesized a series of thiophene and tetrahydrobenzo [*b*]thiophene derivatives as potent and selective CB2 ligands. Comparative analysis showed that compounds bearing 4,5,6,7-tetrahydrobenzo[*b*]thiophene core possessed much more CB2 potency than the compounds with 2-phenylthiophene core, but the selectivity was slightly reduced.^34^
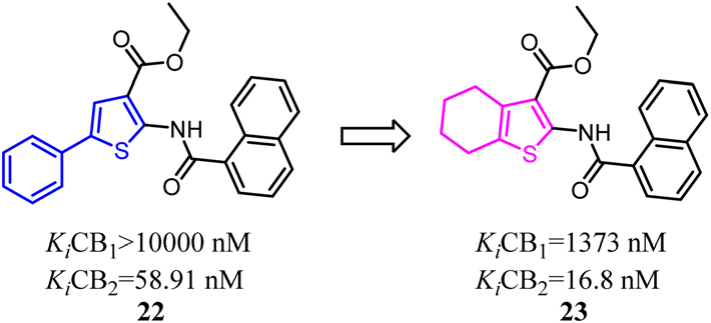


Kusakabe's group previously identified 2-pyridone derivatives as potent CB2 agonists, which had unacceptable pharmacokinetic profiles with no significant effect *in vivo*. To improve these profiles, they conducted further structural optimization based on 24, which formed the bicyclic 2-pyridone compound 25 with improved affinity and selectivity for CB2. 25 inhibited compound 48/80 induced scratching behavior at a dose of 100 mg kg^−1^ in a mouse pruritus model. In addition, the docking model of 25 with an active-state CB2 homology model indicated the structural basis of its high affinity and selectivity over CB1.^35^
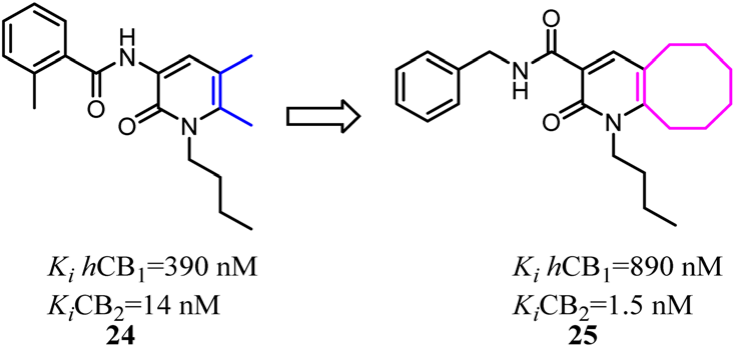


To further explore the pharmacological profiles of tricyclic indenopyrazole scaffold, Pinna's team replaced the benzene ring of the tricyclic indenopyrazole scaffold with a ring-fused thiophene moiety to obtain a novel compound bearing dihydrothienocyclopentapyrazole scaffold that were selective for CB2. The more potent and selective analogues in these compounds possessed binding affinity of 1.1–7.2 nM at CB2 and selectivity over CB1 of up to 485-fold. According to *in vitro* assays, the novel compounds acted as CB2 agonists based on the evaluation of P-ERK 1/2 expression in HL-60 cells.^36^
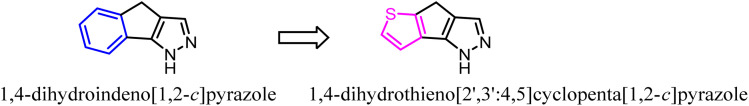


A number of studies have indicated that compound 26 and its derivatives were rapidly metabolized when incubated with human hepatocytes or human liver microsomes *in vitro*,^37,38^ which suggested that the pharmacokinetic profiles of these amidoalkylindoles were not optimal and still had some room for improvement. To improve the pharmacokinetic profiles, Zhang and his co-workers designed a series of 1*H*-pyrazole-3-carboxamide derivatives to overcome the metabolic liability of *N*-alkyl indole-based synthetic cannabinoids while maintaining the major pharmacophoric elements of compound 27. Further structure optimization along with a series of pharmacological evaluations led to the identification of compound 27, which was a potent and selective CB2 agonist with good metabolic stability and favorable pharmacokinetic properties both *in vitro* and *in vivo*.^39^
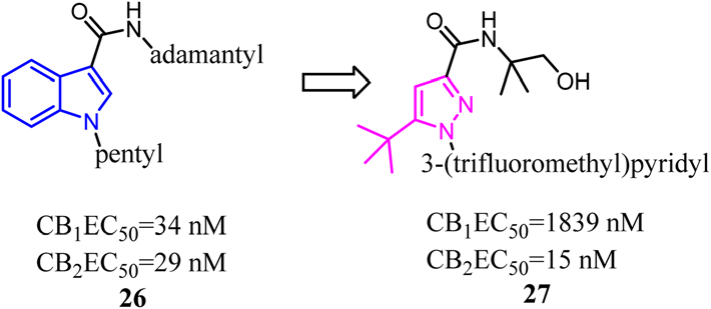


A trend of mono- or bicyclic core bearing one or more heteroatoms such as oxygen or nitrogen, a carbonyl containing linker bound to bulky aliphatic or aromatic groups was generally seen with reported selective CB2 chemotypes.^40^ Osman's group applied structural and core enhancement strategies to construct a novel promising series of potent CB2 agonists. They started from the monocyclic 4-methyl-2-substituted thiazole-5-carboxamides, then explored the effect of core enlargement through condensing a benzene ring to form benzothiazole-2-carboxamides bearing different bulky alicyclic, aromatic and halogenated amide substituents.^41^ These *N*-(3-pentylbenzo [*d*]thiazol-2(3*H*)-ylidene) carboxamide derivatives presented the highest affinity and selectivity for CB2 receptors with *K*_i_ in the picomolar or low nanomolar range, and the selectivity indices (*K*_i_ hCB_1_/*K*_i_ hCB_2_) achieved to 429 folds. Furthermore, these optimized compounds also exhibited the full agonism in cellular assays with EC_50_ in the low nanomolar range. Compound 29 presented remarkable protection against DSS induced acute colitis in mice model.
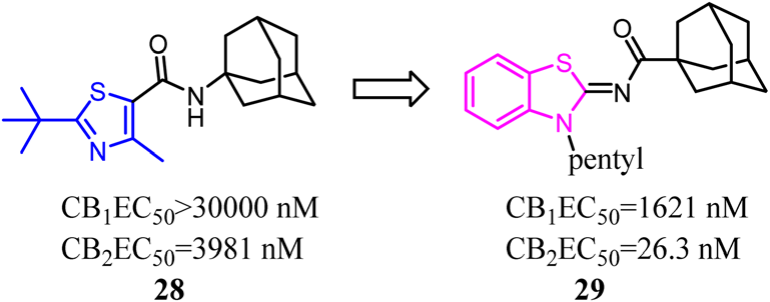


Balancing potency and selectivity with acceptable drug-like properties were very difficult despite an intense exploration of SAR. To achieve the balance point, Hickey's team reduced the seven-member diazocyclic ring to a five-member nitrogen heterocyclic ring(proline). Analogs containing the new proline scaffold exhibited picomolar CB2 activity and high selectivity, and initial drug-like profiling that inspired further optimization within the series.^30^
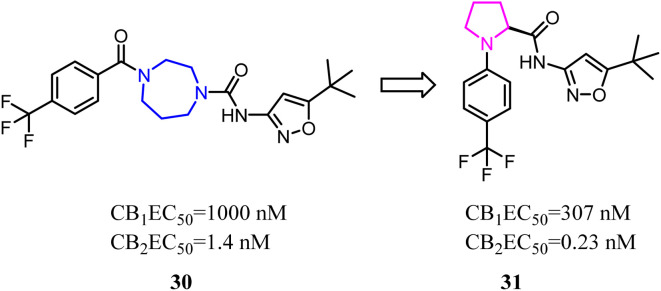


Compound 32 was a potent and selective CB2 ligand with acceptable pharmacokinetic parameters. To further explore the SAR of compound 32, Tong's group replaced the ring A and ring B respectively with indole ring and piperidine ring.^42^ The scaffold hopping strategy improved the binding affinity and selectivity for CB2.
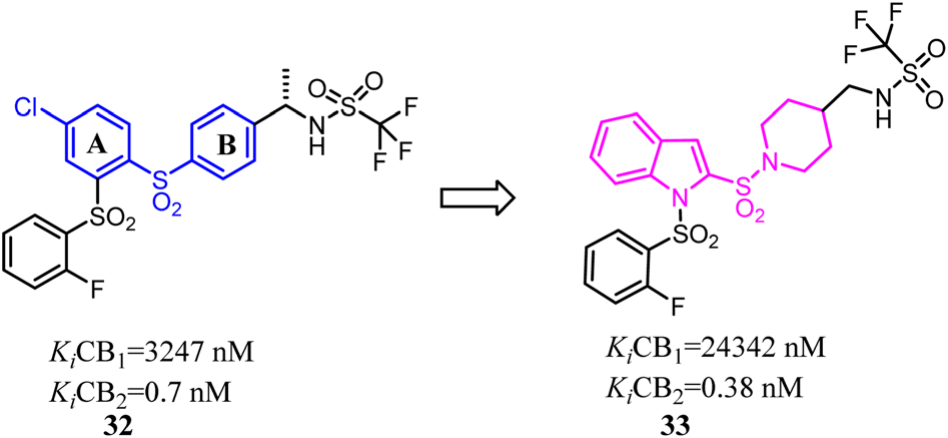


Müller's team discovered compound 34, which belonged to 3-benzyl5-methoxycoumarin derivatives and could interact with both CB1 and CB2. To improve the selectivity towards CB2, the main change was that the aromatic C ring was removed and benzyl group was introduced to form 7-alkyl-3-benzylcoumarins. These novel compounds were tested at cannabinoid CB1 and CB2 in radioligand binding and cAMP accumulation assays. Among them, 35 was a selective CB2 agonist.^43^
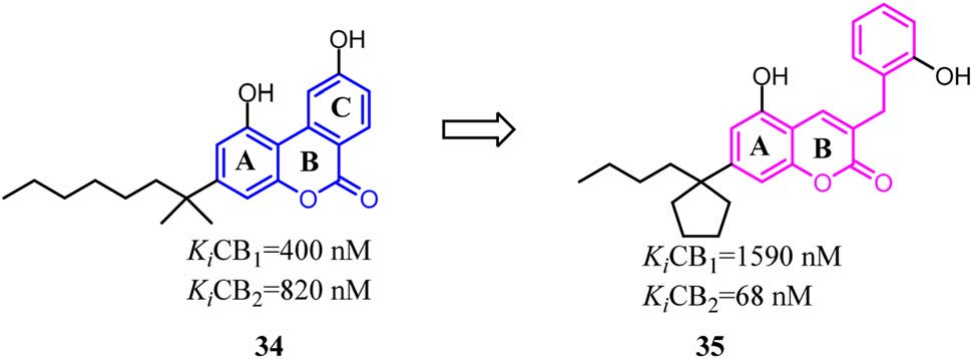


Compound 36 as a both CB1 and CB2 ligand belonged to 1,2-dihydro-2-oxo-pyridine-3-carboxamide derivatives. To improve the selectivity for CB2, Manera's team conducted the modification and optimization by insertion of an aryl moiety at the 4-position of the 1,2-dihydro-2-oxo-pyridine ring to obtain 4-substituted or 4,5-disubstituted-1,2-dihydro-2-oxo-pyridine-3-carboxamide derivatives. Generally, all ligands tended to show higher affinity for CB2, with a selectivity factor in the range of 2–54.^44^ To avoid the CNS side effect, Manera's team applied the retro-amides strategy to develop and synthesize the positive allosteric modulator that targets CB2 based their previous works. Finally, compound 38 displayed antinociceptive activity *in vivo* in an experimental mouse model of neuropathic pain.^45^
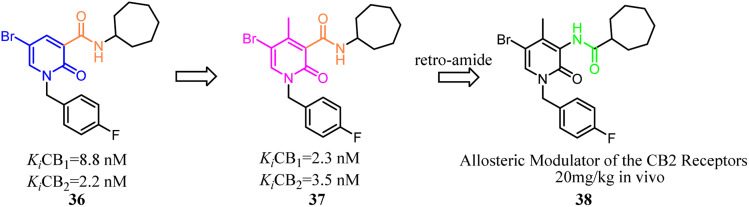


Kusakabe's group identified compound 39 as a potent and selective CB2 agonist *via* high-throughput screening. However, it was difficult to synthesize due to its two asymmetric carbons, which made it difficult to further explore the efficient SAR. Furthermore, the isoquinolone scaffold was generally highly lipophilic, which was disadvantageous to drug-likeness. To overcome these two major problems, they designed a 2-pyridone-based scaffold in order to remove the two asymmetric carbons and reduce lipophilicity in isoquinolone 39. The result showed that 2-pyridone-based lead compound 40 exhibited moderate affinity for CB2. Optimization of 40 led to compound 41 with high affinity and selectivity for CB2.^46^
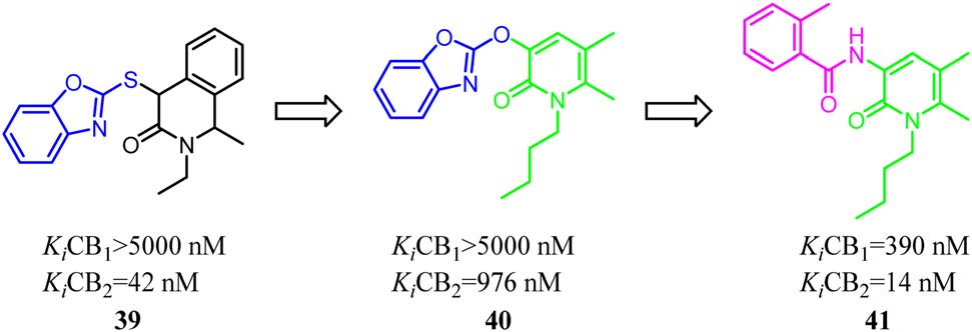


Pinna' group developed a series of tricyclic derivatives as potent CB2 ligands. Among them, tricyclic 4,5-dihydro-1*H*-benzo[*g*]indazole compounds showed lower activity and selectivity toward CB2. To improve the affinity and selectivity, they used the 1,4-dihydroindeno[1,2-*c*] pyrazole scaffold to replace 4,5-dihydro-1*H*-benzo[*g*]indazole. Significantly, after optimization, four compounds were highly selective for the CB2 receptor and were also subjected to GTPgS binding analysis showing antagonist/inverse agonist properties.^47^
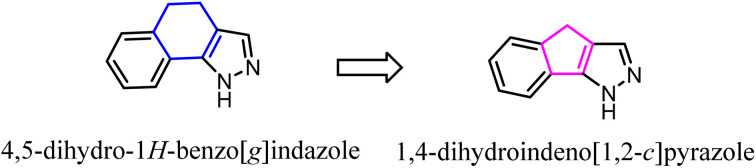


Jagerovic's team synthesized a selective CB2 ligand compound 42(*K*_i_ CB1/*K*_i_ CB2 = 258) which was derived from SR144,528. To further improve the selectivity and affinity for CB2, they expanded the pyrrole five-membered ring of compound 42 to pyridazin-3(2*H*)-one six-membered ring and formed compound 43(*K*_i_ CB1/*K*_i_ CB2 > 2000) with antagonism.^48^
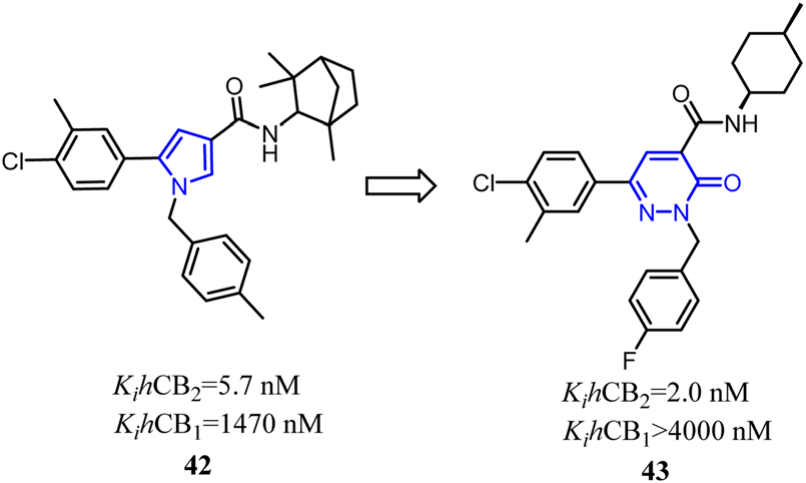


### 2.3 Comformational restriction

One of the commonly used strategies in drug design to increase affinity and selectivity of a given “flexible” lead for its pharmacological target was to conformationally constrain it to mimic the so-called bioactive conformation.

Because of the promising activity of a series of 4-oxo-1,4-dihydroquinoline-3-carboxamide, Millet's team developed constrained with the improved affinity for CB2 and high selectivity over the CB1 analogues based on a 2*H*-pyrazolo[4,3-*c*]quinolin-3(5*H*)-one scaffold. Among them, compound 45 was found to protect mice against experimental colitis after oral administration.^49^
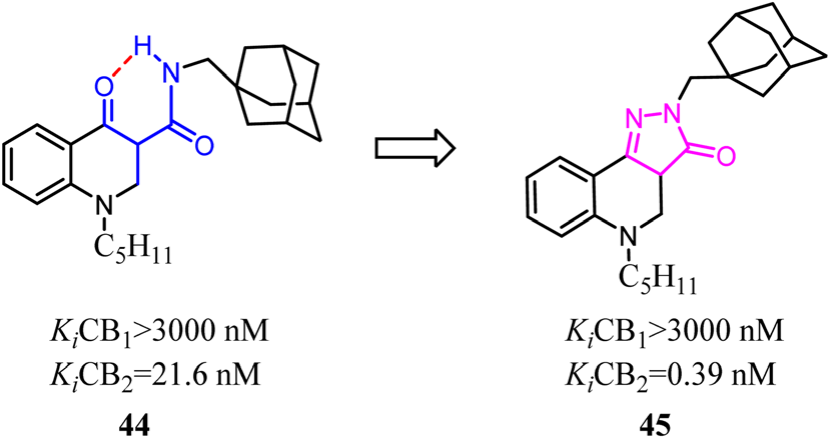


Nettekoven's team conducted a conformational analysis of the most active derivatives revealed the replacement of the anthranilic acid ester moiety with bi-cyclic systems. A series of highly potent and selective adamantane CB2 agonists were identified *via* high-throughput screen. The novel SAR of the designed compounds was established and physicochemical properties were markedly improved.^50^
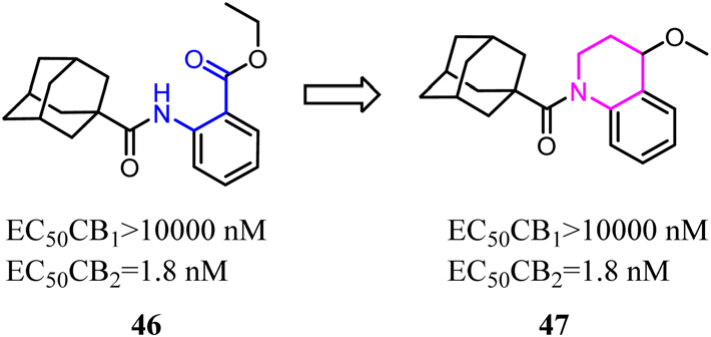


Various reported 5-membered heterocyles substituted by *tert*-butyl and cyclopropylmethyl groups were the selective CB2 agonists. To improve CB2 potency and microsomal stability, Han's group designed 3-5-5 fused tricycles *via* an intramolecular constraint. Then they performed further structural optimization that novel 1a,2,5,5a-tetrahydro-1*H*-2,3-diaza-cyclopropa[*a*]pentalen-4-carboxamide CB2 selective ligands for the potential treatment of pain was described.^51^ Compound 50 had good balance between CB2 agonist potency and selectivity, and possessed overall favorable pharmaceutical properties.
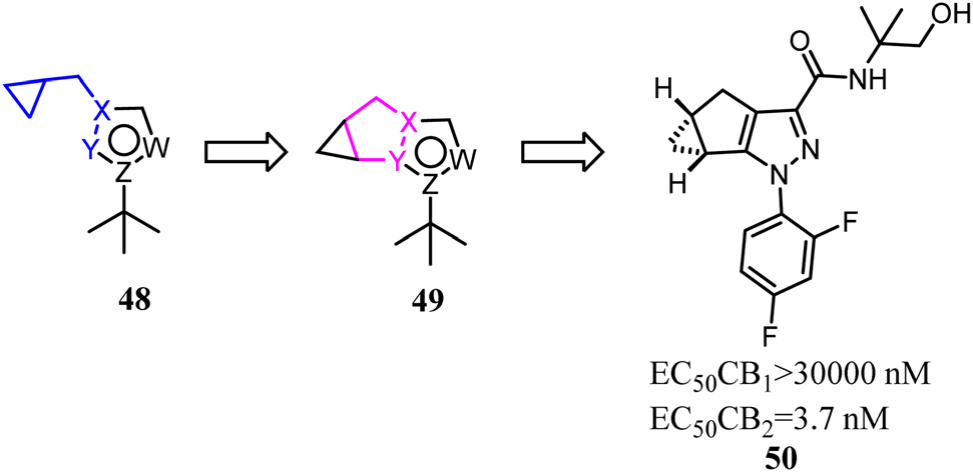


Mugnaini and his co-workers designed and synthesized the 6-member ring merged thiophene based on the substitution on the 4 and 5 position on thiophene, which restricted the hole scaffold.^52^ Most of the optimized compounds possessed high CB2 affinity at low nanomolar concentration, good receptor selectivity, and agonistic functional activity. Especially the full agonist 52, exhibiting the best balance between receptor affinity and selectivity, was tested *in vitro* in an experimental model of allergic contact dermatitis. The result showed that it could block the release of MCP-2 in HaCaT cells at 10 mM concentration.
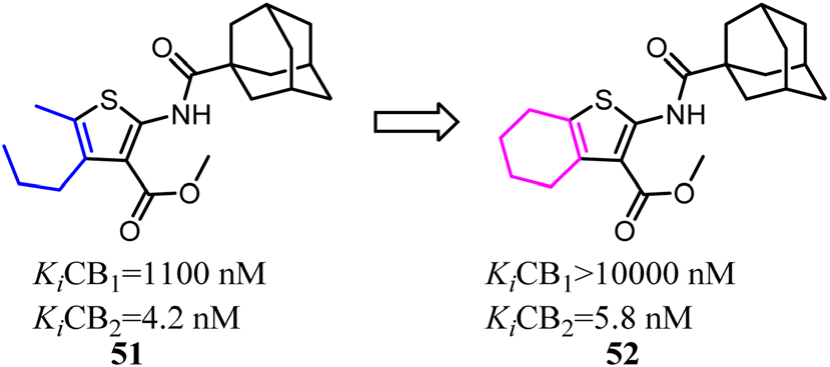


Kassiou' team studied the structure–activity relationship of pyrazolidene benzamide CB2 agonists suggested the -ylidene benzamide moiety was crucial for functional activity at the CB2. They used 1,2,4-triazine to replace the amide linkage moieties between the pyrazole and substituted phenyl group, which restricted the conformation of whole scaffold. Among them, the identified pyrazolo-[2,3-*e*]-[1,2,4]-triazine agonist 54 was potent and selective. Docking studies had revealed key structural features of the linkage group that were important for potent functional activity.^53^
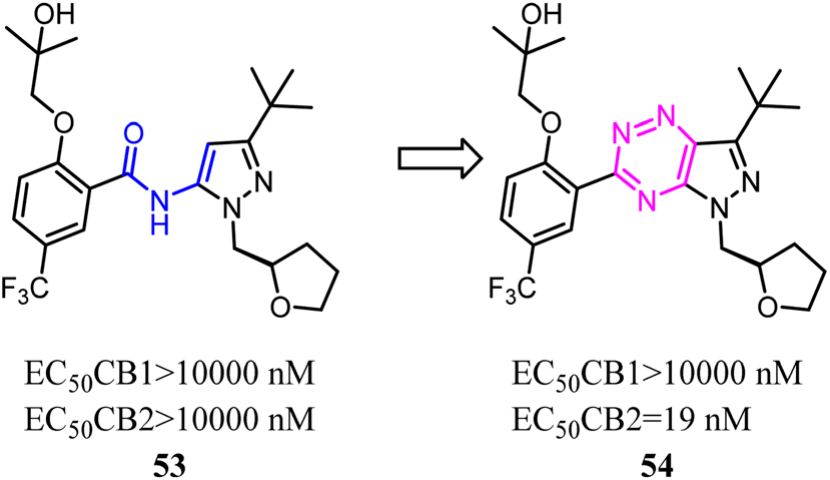


Corelli's team reduced the conformational freedom of lead compound 55, which they removed the linker between the aromatic rings of 55 in order to obtain 5-(hetero)aryl substituted indole 56.^54^ Most of the compounds exhibited affinity for CB2 in the nanomolar range, with *K*_i_ values spanning 3 orders of magnitude (377–0.37 nM), and moderate to good selectivity over CB1.
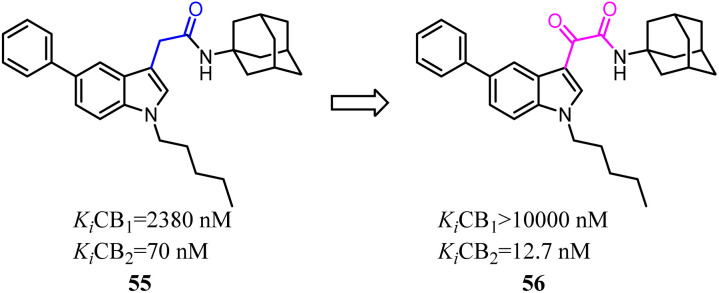


### 2.4 Bioisosterism

Bioisosterism strategies were widely used in drug design, such as kinase inhibitors and G protein-coupled receptor ligands.^55,56^ Many examples of CB2 ligand design *via* bioisosterism strategy were reported. Millet's team synthesized and characterized ferrocene CB2 ligands and fatty acid amide hydrolase inhibitors based on the bioisosterism strategy, which the adamantyl amine was replaced with aminoferrocene. The obtained bio-organometallic isoxazoles were assayed for their effects on CB1 and CB2 receptors as well as on fatty acid amide hydrolase. None had any fatty acid amide hydrolase activity but compound 58, was found to be a potent CB2 ligand (*K*_i_ = 32.5 nM).^57^
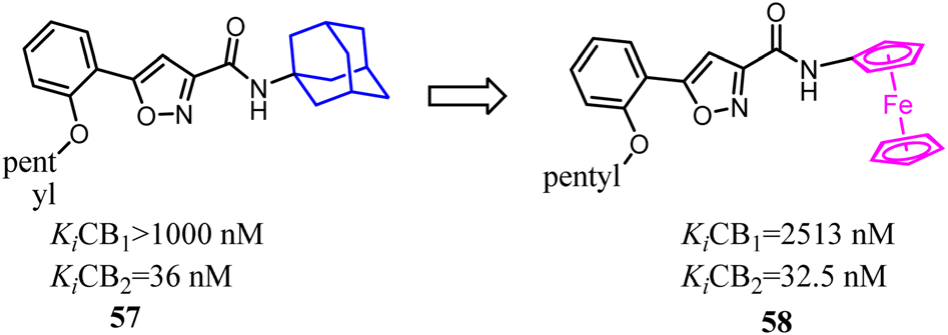


Previously Yu's team identified a series of amidoalkylindoles as potent and selective CB2 partial agonists.^58^ However, the compound 59 suffered from limited aqueous solubility and high lipophilicity. Then they reported their continuous effort to improve the aqueous solubility by introducing N atom to the amidoalkylindole framework. Synthesis, characterization, and pharmacology evaluations were described. Bioisosteric replacements of the indole core with an indazole, azaindole and benzimidazole were explored. Benzimidazole 60 was a potent and selective CB2 partial agonist with improved aqueous solubility.^59^
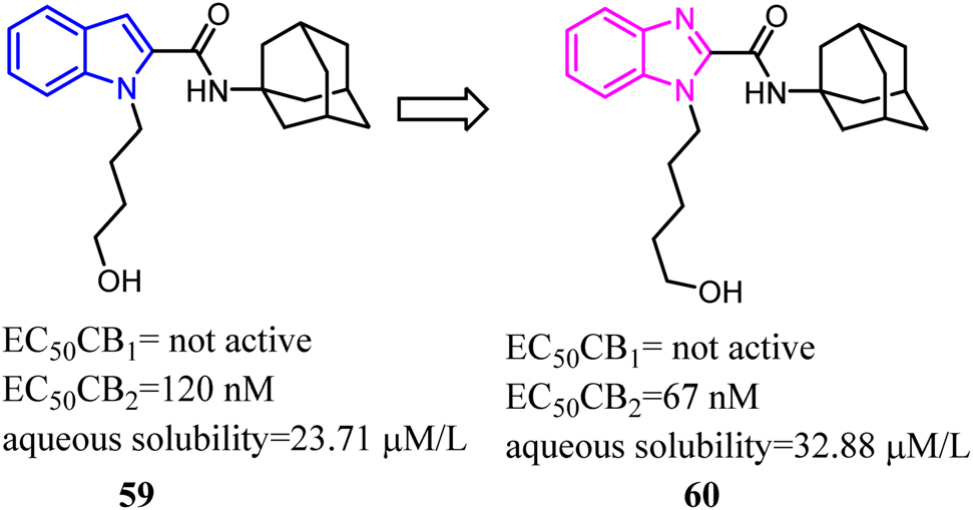


In recent years, there had been a continuous interest in the development of cannabinoid receptor ligands that may serve as therapeutic agents and/or as experimental tools.^60^ This prompted researchers to design and synthesize analogues of the CB2 antagonist (SR144528). The structural modifications involved the bioisosteric replacement of the pyrazole ring by a pyrrole ring and variations on the amine carbamoyl substituents. The fenchyl pyrrole analogue 62 did not only exhibit high affinity with selectivity for the CB2 but also worked as antagonists/inverse agonists in [35S]-GTPgS binding assay and functional bioassay *in vitro*.
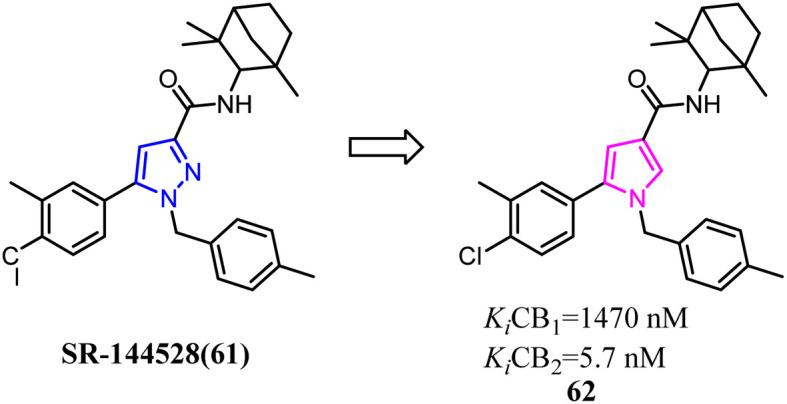


Corelli's team selected the heterocyclic systems as potential bio-isosteres of the amide linker for a series of 1,6-disubstituted-4-quinolone-3-carboxamides, which were the potent and selective CB2 ligands that exhibited poor water solubility, with the aim of improving their physicochemical profile and also of clarifying properties of importance for amide bond mimicry.^61^ Among them, a new compound 64 emerged as the most promising in terms of both physicochemical and pharmacodynamic properties.
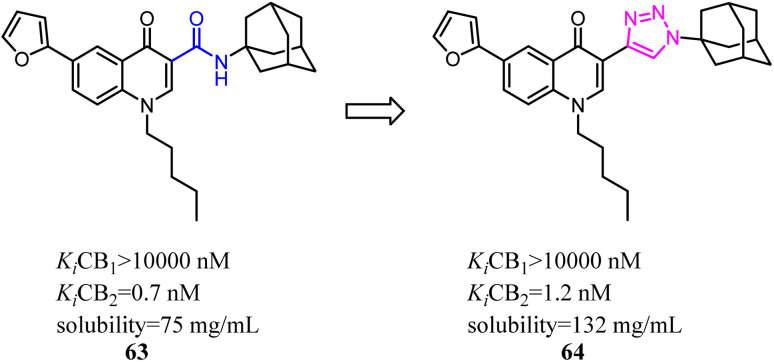


With the aim of improving the selectivity of indole derivatives for CB2, Kassiou' group designed and synthesized potent and selective CB2 agonists based on a simple structural modification, bioisosteric replacement of the terminal –OH of N-1 side chain with methyl. They used potent, non-selective synthetic cannabinoids designer drugs to develop selective CB2 receptor agonists, which was efficient and feasible.^62^ Among them, 66 was found to be a potent and selective agonist with favorable physicochemical properties.
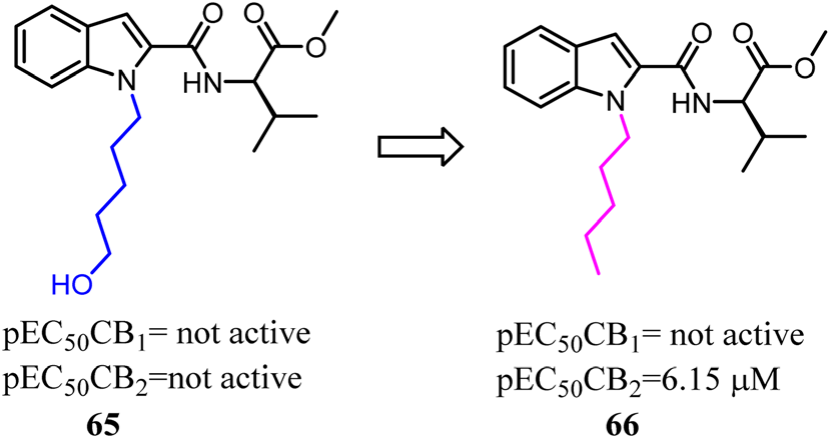


Synthetic cannabinoid designer drugs featuring bioisosteric fluorine substitution were identified by forensic chemists and toxicologists with increasing frequency.^63^ Terminal fluorination of *N*-pentyl indole synthetic cannabinoid compound was sometimes known to improve cannabinoid receptor binding affinity. This study explored the functional activities of synthetic cannabinoid designer drugs JWH-018, UR-144, PB-22, and APICA, and their respective terminally fluorinated analogues AM-2201, XLR-11, 5F-PB-22, and STS-135 at human CB1 and CB2 receptors *in vitro via* using a FLIPR membrane potential assay. All compounds demonstrated agonist activity at CB1 (EC_50_ = 2.8–1959 nM) and CB2 (EC_50_ = 6.5–206 nM) receptors. Unfortunately, the selectivity for CB2 was not improved.
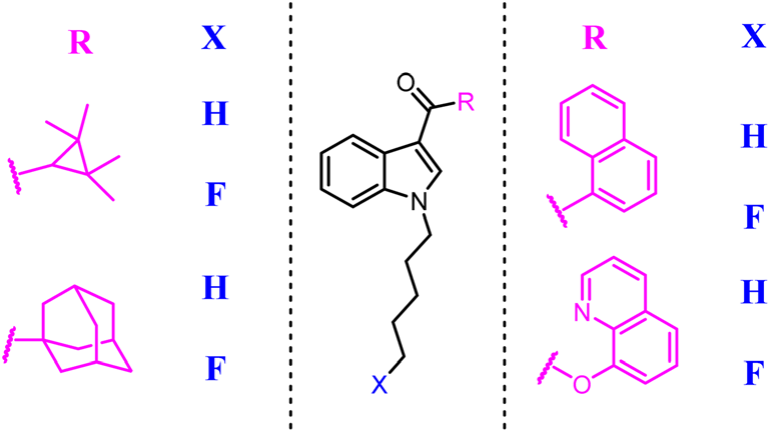


Moore's team refined the structure activity relationship of the 2,6-dihydroxy-biphenyl-aryl-methanone scaffold as the CB2 inverse agonists. A series of compound 68 derivatives were synthesized and measured for affinity/selectivity, potency, and efficacy in regulating cAMP production and β-arrestin recruitment, and the design of all derivatives based on the replacement A ring with thiophene. Compound 68 demonstrated a significant increase in potency and efficacy for cAMP stimulation compared to 67. This compound was highly efficacious in biasing microglia to an M2 pro-wound healing phenotype in LPS stimulated cell lines.^64^
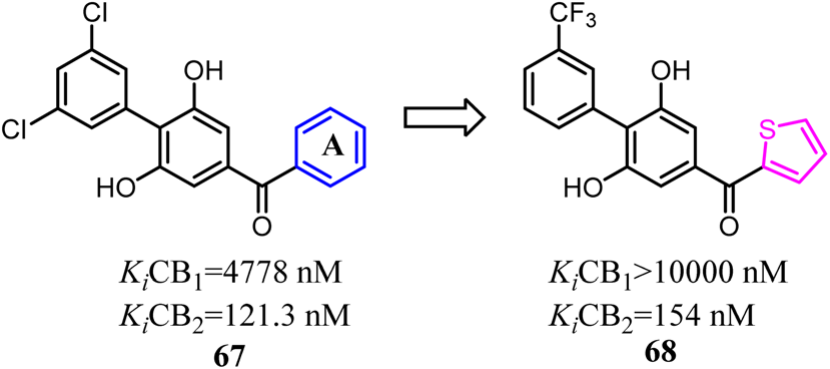


Manera's team used their previously reported series of 1,8-naphthyridin-2(1*H*)-on-3-carboxamides as lead compounds, in order to evaluate the effects of the modification of the 1,8-naphthyridin-2(1*H*)-one ring system, the 1,8-naphthyridin-2-one core was replaced with quinolin-2-one core. The obtained results indicated that the novel series of quinolin-2(1*H*)-one-3-carboxamides have interesting effects on the CB2 affinity over CB1. Furthermore, the newly synthesized CB2 ligands inhibited proliferation of several cancer cell lines.^65^
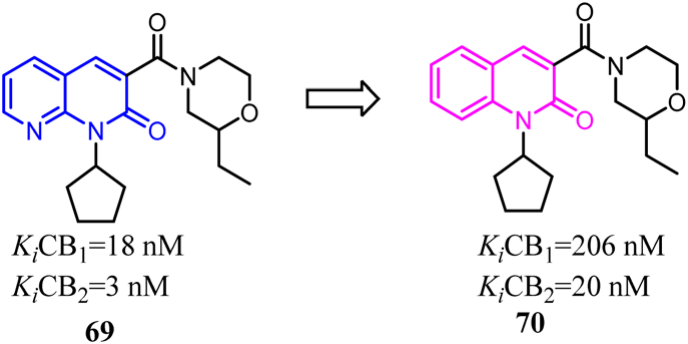


The identified thienopyrimidine 71 was a CB2 agonist with moderate selectivity over the CB1 receptor, but suffered from poor *in vitro* metabolic stability and high *in vivo* clearance.^66^ To overcome metabolic and clearance problems, Hollinshead's team conducted the optimization and modification based on replacement of the thienopyrimidine with a novel more polar series of purine. Most compounds from this new scaffold showed great potency and excellent selectivity for CB2, and were also fully efficacious agonists of the human CB2. Compound 73 was a centrally penetrant molecule which possessed good biopharmaceutical properties, was highly water-soluble, and demonstrated robust oral activity in rodent models of joint pain.
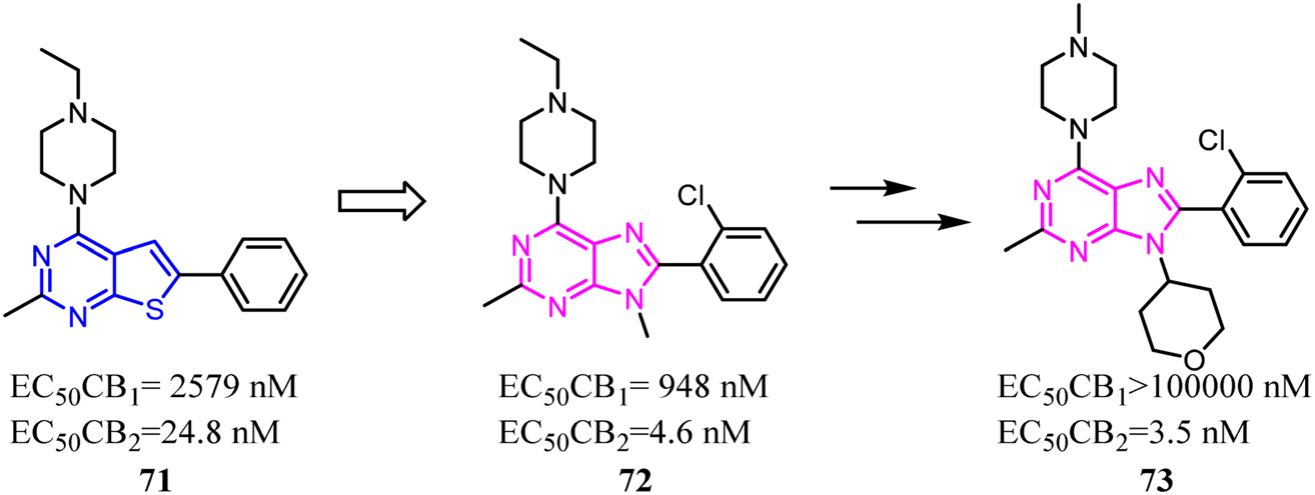


Mu's team evaluated 74 as an imaging agent for CB2, and it was not optimal yet probably due to its high lipophilicity.^67^ To search for suitable candidate compounds that retained the high affinity and selectivity profile of 74 for CB2 but showed reduced lipophilicity. They designed and synthesized a series of novel 4-oxo-quinoline derivatives based on the lead structure of 74, which the *n*-pentyl side chain was replaced with oxyalkyl chain. Compared to 74, 75 exhibited a higher binding affinity towards CB2 with a selectivity over CB1 and lower lipophilicity. 75 was stable *in vitro* in rodent and human plasma over 40 min, whereas 47% intact compound was found *in vivo* in rat blood plasma 20 min post injection (p.i.). Based on the studies of structure–activity relationship, compound 75 as a very promising ligand was selected for radiolabeling with carbon-11 and as an imaging agent was examined *in vitro*/*in vivo* studies.^68^
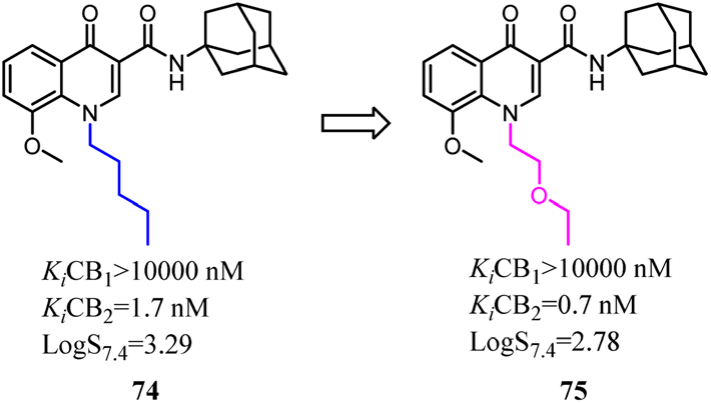


Tabrizi's team reported a novel series of 7-oxopyrazolo[1,5*a*]pyrimidine-6-carboxamides that were found to be potent and selective CB2 receptor ligands. To conduct a pharmacophore exploration and optimization effort around the heteroaryl central scaffold, the [1,2,4]triazolo[5,1-*b*]pyrimidin-7-one core was replaced with pyrazolo[5,1-*b*]pyrimidin-7-one core.^69^ All of the new compounds showed high affinity and selectivity for the CB2 in the nanomolar range. In cAMP assays, the novel series showed stimulatory effects on forskolin-induced cAMP production acting as inverse agonists.
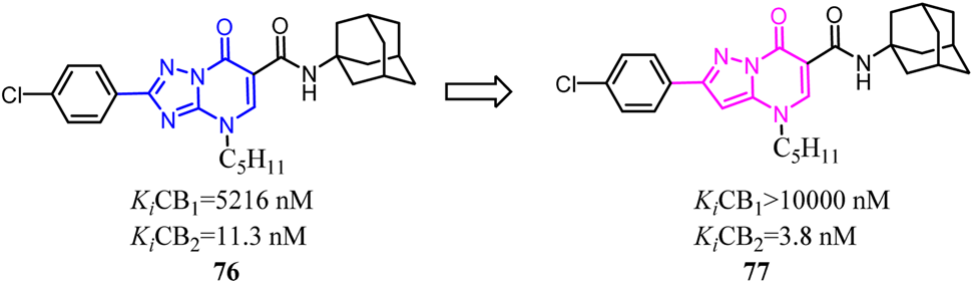


Millet's group designed, synthesized a new series of 3-carboxamido-5-aryl-isoxazoles, based on the structure of SR144,528*via* bioisosteres strategy. The pyrazole and 1,3,3-trimethylbicyclo[2.2.1]heptane of SR144,528 were replaced respectively with isoxazole and adamantane, which formed the compound 78. The pharmacological results had identified the compound 78 as the great selective CB2 agonist with *in vivo* anti-inflammatory activity in a DSS-induced acuted colitis mouse model.^70^
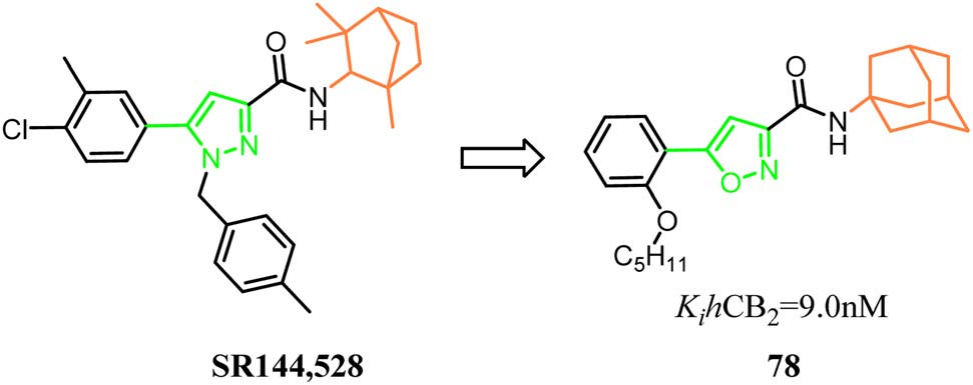


79 was a nonselective CB2 ligand containing cannabinoid scaffold, and the *K*_i_(CB_2_)/*K*_i_(CB_1_) selectivity ratio was 3.1. Jagerovic' group developed the selective CB2 ligand compound 80 based on compound 79, which the pyrazole ring was replaced with isoxazole ring. Finally, the *K*_i_(CB_2_)/*K*_i_(CB_1_) selectivity ratio was raised above 3100, and compound 80 did not exhibit agonism *via* cAMP assay(CB2 CI_50_ = 4.2 nM) and [^35^S]-GTPγS assays(CB2 CI_50_ = 38.6 nM), but also dampened neuroinflammation.^18^
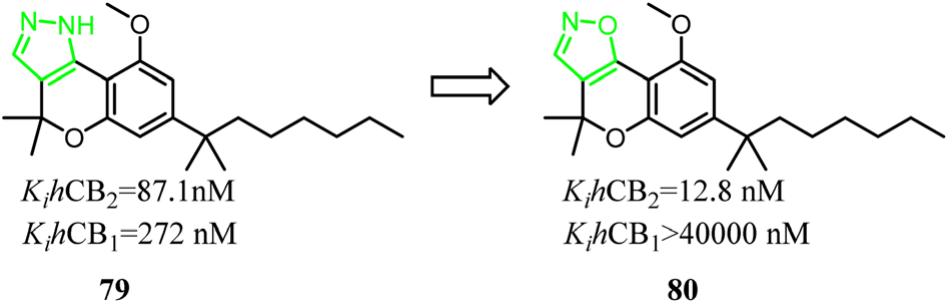


### 2.5 Side chain modification

Side chains were the significantly and specially pharmacophores against CB2, which their geometrical shape and physicochemical property would affect the potential and selectivity of the ligands to CB2.

Δ8-THC was a nonselective CB2 ligand and the *K*_i_(CB_2_)/*K*_i_(CB_1_) selectivity ratio was 1. Huffman's group synthesized the first selective CB2 ligand JWH-133 by changing the side chain of Δ8-THC.^17^ The *K*_i_(CB_2_)/*K*_i_(CB_1_) selectivity ratio was raised to 199 and this trend of high selectivity ratio was demonstrated in SAR study. JWH-229 was the selective CB2 ligand and the *K*_i_(CB_2_)/*K*_i_(CB_1_) selectivity ratio was 174.^71^
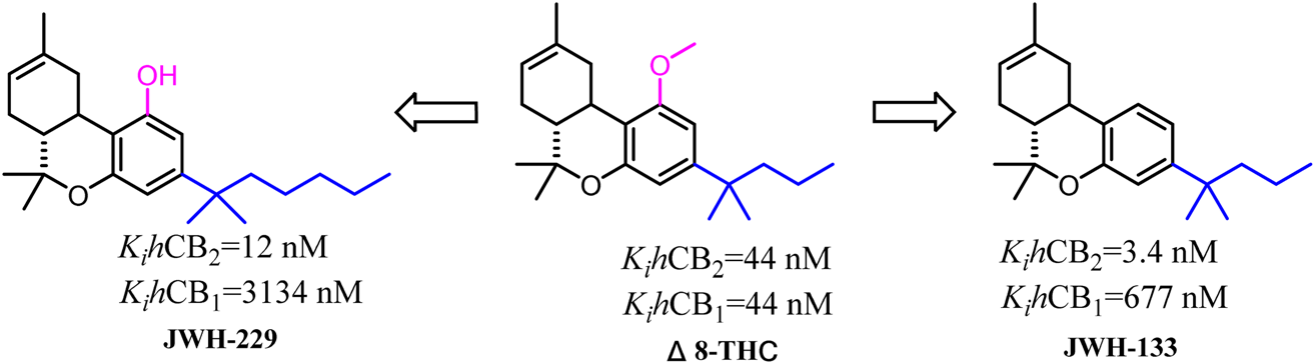


Huffman's group also developed indole scaffold selective CB2 ligands including JWH-151. These compounds were derived from WIN-55,212-2 which was a nonselective CB2 ligand and was always used as a tool compound. The key optimization happened on N-1 postion such as the substituted *N*-ethylmorpholine was replaced with propyl group which formed the most potent CB2 ligand JWH-151 with full agonism.^72^
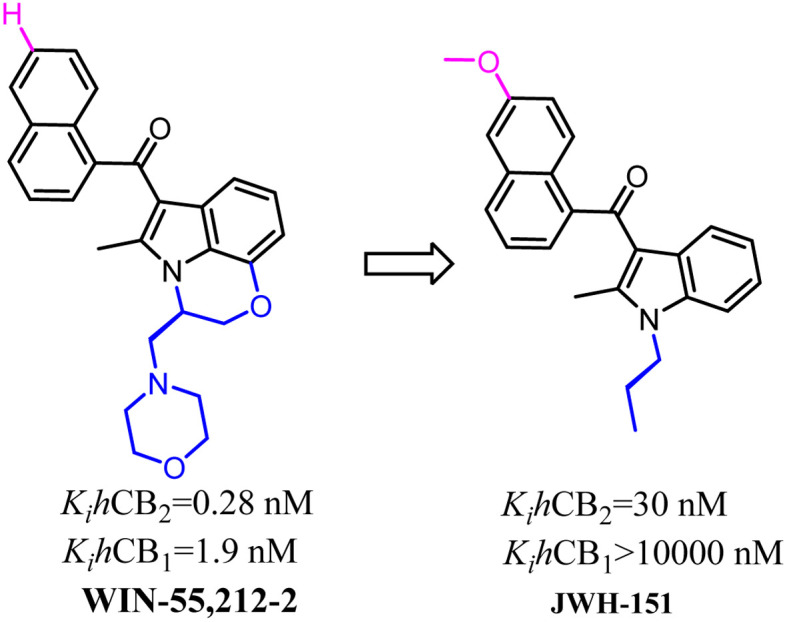


Compound 81 was highly potent and fully efficacious CB2 agonists with a selectivity over the CB1 receptor of 1300 fold. However, the compound possessed low solubility, a moderate stability of 110 min in human liver microsomes and a moderate to low stability of 81 and 2 min in rat liver microsomes. To remove this major barrier, Riether's team replaced the 4-(trifluoromethyl) phenyl with tetrahydro-2*H*-pyranyl, and formed compound 82 which was a potent CB2 agonist with high selectivity over the CB1 and good pharmacokinetic properties.^73^
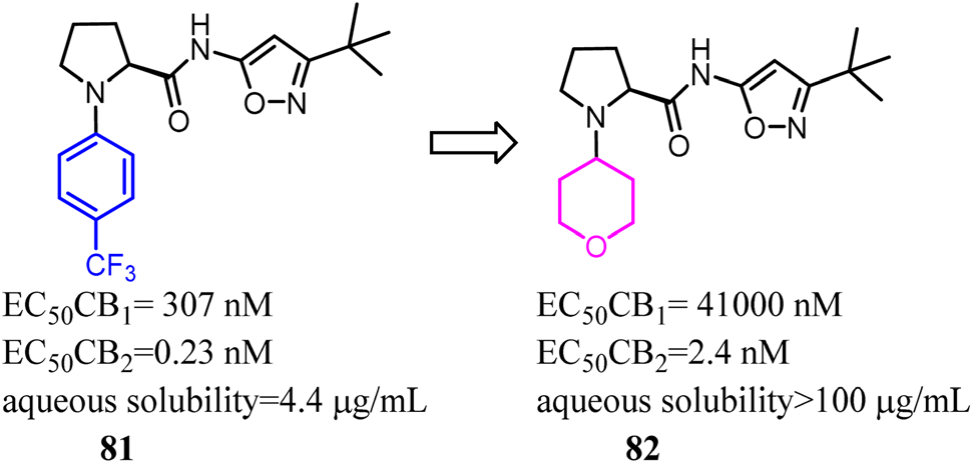


Compound 83 was the first synthesized compound in the piperidine class, demonstrated potency and full efficacy as a CB2 agonist. As 83 possessed low solubility, less than desirable metabolic stability in human liver microsomes and an insufficient CB2 *versus* CB1 selectivity window. Nevertheless, Bartolozzi's team replaced 3-(trifluoromethyl) pyridyl with thiomorpholine-4-carbonyl in the N-1 side chain, which displayed selectivity over CB1 and acceptable drug like properties. Especially in rats, compound 84 demonstrated a favorable pharmacokinetic profile and efficacy in a streptozotocin-induced diabetic neuropathy model, with full reversal of mechanical hyperalgesia.^74^
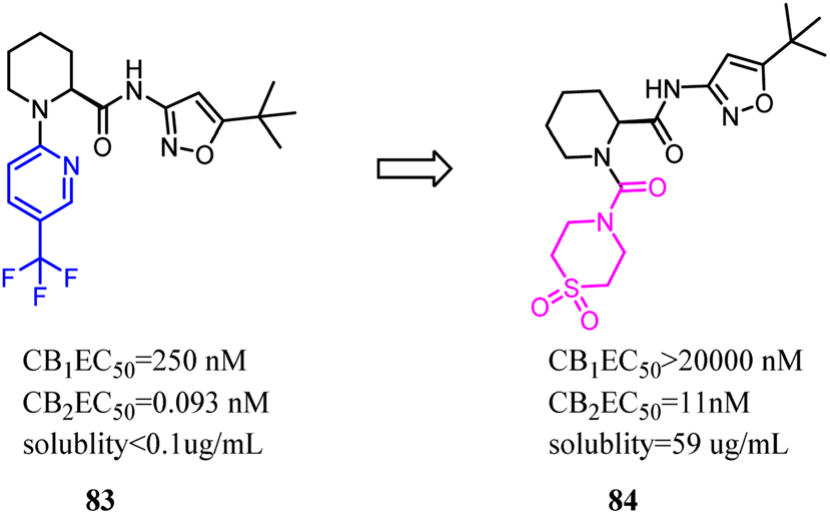


85 was a CB2 PET tracer with a high CB2 affinity and excellent selectivity over the CB1, however, it also exhibited some disadvantages like a rapid metabolism and relatively high lipophilicity.^70,73–75^ To inhibit oxidative degradation by CYP enzymes, Wünsch's team conducted a little bit of significant modification that introduction of an additional methyl moiety into the fluoroethyl side chain of 85 resulted in the fluoroisopropyl derivatives 86 with almost the same CB2 affinity and selectivity over the CB1. Both compounds 85 and 86 had same CB2 affinity, but compared to 85, the metabolic stability of compound 86 was increased through the subsequent LC-MS-MS analysis. It could be concluded that compound 86 did not only maintain the CB2 affinity, but was able to shield the carbazole N-atom from oxidative attack by microsomal CYP enzymes.^76^
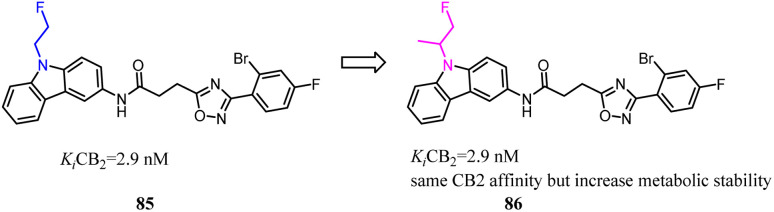


4-Quinolone-3-carboxamide derivatives possessed high affinity and selectivity against CB2 and characterized by different functional profiles. However, these compounds mostly exhibited high *C*log *P* values and low water solubility in excellent *in vitro* profile.^77^ With the aim to improve their physicochemical properties and aqueous solubility, a simple substitution at the terminus of alkyl chain with a substituted N. After optimization, the obtained compound 88 slightly reduced receptor affinity compared to the lead compound 87, but greatly enhanced solubility.
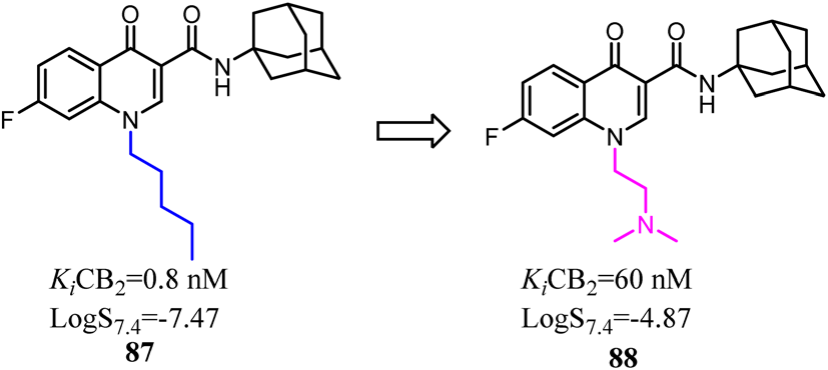


To further explore the affinity and selectivity of 1,8-naphthyridine and quinoline derivatives which belonged to CB2 ligands, Manera's team designed and synthesized a series of novel quinoline-2(1*H*)-one- and 4-hydroxy-2-oxo-1,2-dihydro-1,8-naphthyridine derivatives with high CB2 receptor affinity and selectivity. The SAR study revealed that 4-F-benzyl was replaced with *n*-pentyl as a side chain could increase the both affinity and selectivity for CB2. Obtained compound 90 behaved as a partial agonist towards CB2 and induced a concentration-dependent decrease of cell viability on LNCaP, a prostatic cancer cell line expressing CB2 receptor.^78^
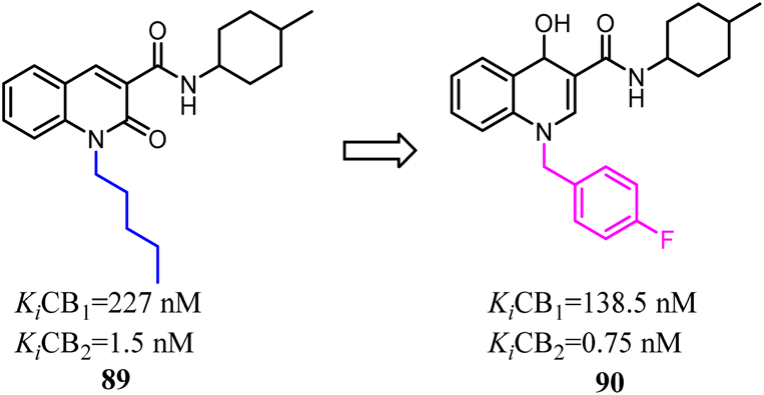


Murineddu's team had developed a series of pyridazinone-based derivatives, which endowed potent affinity and high selectivity towards CB2. Among these novel compounds, 91 exhibited high CB2 affinity and notable selectivity (*K*_i_ CB1/*K*_i_ CB2 > 2000). To further explore structure–activity relationships of the these new class CB2R ligands,^79^*n*-pentyl was replaced with 4-F-benzyl as a side chain could increase the both affinity and selectivity for CB2. 92 showed high CB2 affinity, with the *K*_i_ value of 1.6 nM. In addition, functional assays of the compound revealed their pharmacological profiles as CB2 inverse agonists. Compound 92 displayed the highest CB2 selectivity and potency, presenting a favorable *in silico* pharmacokinetic profile.
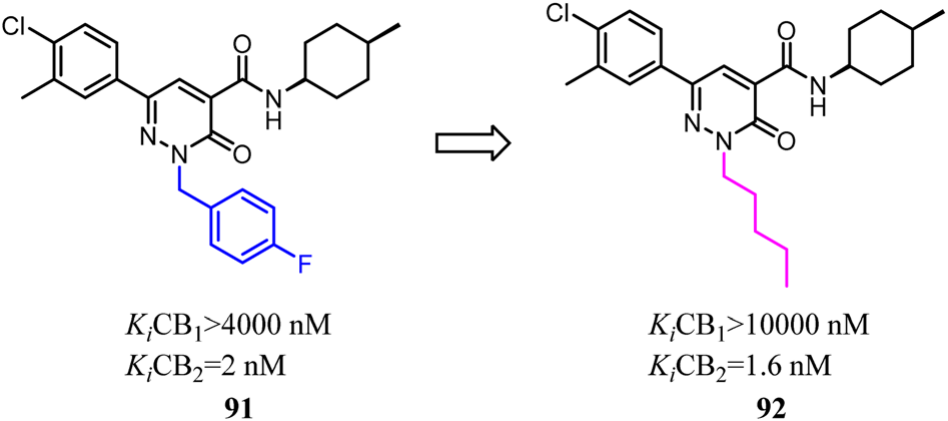


Compound 93 was a potent and selective cannabinoid CB2 agonist. This compound was found to be metabolically unstable, which resulted in low oral bioavailability in rat and endowed off-target activity at the hERG ion channel. To solve these problems, Hermkens's team conducted the optimization of pharmacokinetic properties and hERG affinity, which systematically modulated lipophilicity and basicity. The most significantly optimization happened on the side chains, which 1-piperidinemethylene was replaced with 4-thiomorpholinemethylene 1,1-dioxide and isopropyl methyl was replaced with cyclopropyl. After optimization, the physicochemical properties of 1-(4-(pyridin-2-yl)benzyl)imidazolidine-2,4-dione derivatives were benefit to druggability. Among them, compound 94 was a potent, selective, and orally bioavailable cannabinoid CB2 agonist and was active in a rat spinal nerve ligation model of neuropathic pain.^80^
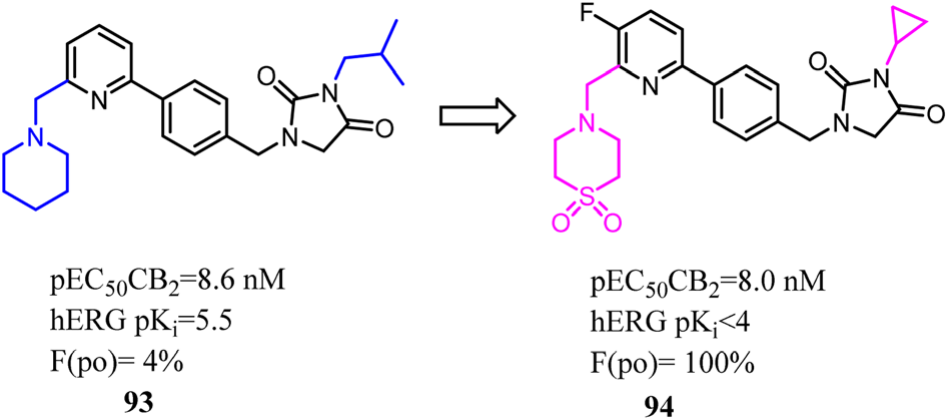


The azaindole nucleus was another structural element which played a significant role in improving affinity and activity at CBs. Azaindoles endowed optimal physicochemical properties because of the combination of an electron-deficient pyridine ring fused with a pyrrole ring, putatively leading to advantages in druglikeness and bioavailability.^81^ Unfortunately, these series compounds were nonselective towards CB2. Crooks's team conducted a side chain optimization of the N-1 in azaindoles, which 4-morpholineethyl was replaced with benzly, and the *K*_i_ values determined from full competition binding curves showed that compound 96 exhibited high affinity and moderate selectivity for CB2.^82^
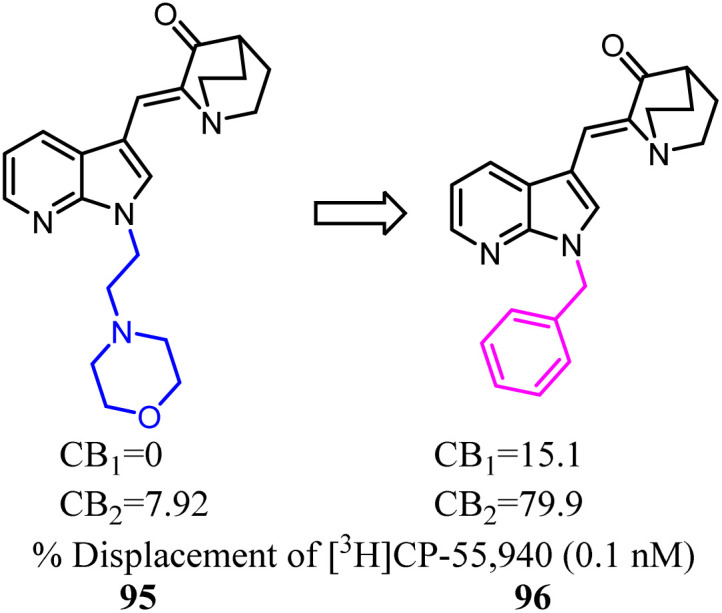


Despite CB2 ligands had a wide range of applications in neuroinflammatory processes, a suitable CB2-targeted probe was currently lacking in clinical routine. Ametamey's team developed the compound 97 which belonged to fluorinated pyridine derivatives, and tested their binding affinities towards CB2 and CB1. Among all the newly designed compound 98 exhibited the highest selectivity index of >12 000, which because the hydroxymethyl was introduced to the cyclopropyl as the side chain. With this remarkable affinity and selectivity, 98 exhibited the most appropriate *in vitro* properties for evaluation as a potential CB2 PET radioligand. Hence, this structure was selected for advanced profiling, radiolabeling, and biological assessment.^83^
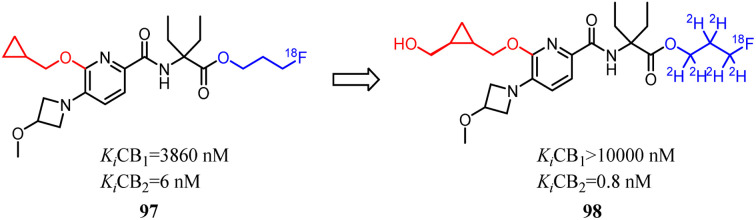


Li and their co-workers designed, synthesized and biologically evaluated a series of indole derivatives with *N*-ethyl morpholine moieties, which were novel CB2 agonists with high efficacy and selectivity. The *N*-ethyl morpholine moiety was moved from N-1 to C-3, the EC_50_ was improved significantly, which the target molecule exhibited high CB2 affinity at low nanomolar concentrations with good receptor selectivity. More importantly, compound 100, the most active compound, had a potent anti-inflammatory pain effect within 12 hours after administration, in a rat model for CFA-induced inflammatory hyperalgesia. Compound 100 had a dose-dependent reversal for hyperalgesia with an estimated ED_50_ value of 1.097 mg kg^−1^. Moreover, compound 96 significantly suppressed the pro-inflammatory cytokines (IL-1β, IL-6 and TNF-α) in CFA-induced lesions.^84^
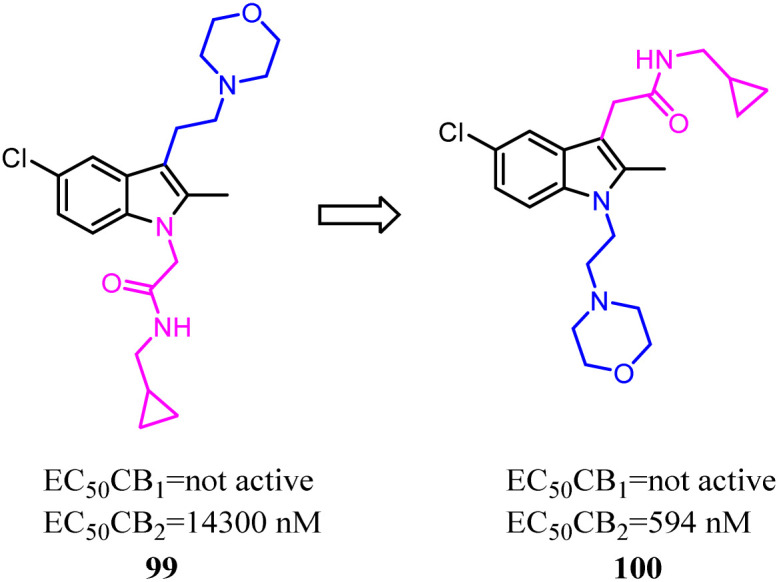


Some studies indicated that CB2 agonists could induce internalization and desensitization of the receptor leading to a decrease in signalling and surface receptor levels.^85^ Positive allosteric modulators represented a promising approach to achieve the potential therapeutic benefits of orthosteric CB2 agonists limiting their adverse effects.^86^ These attempted researchers to develop the first small synthetic CB2 positive allosteric modulator 101, one of the 2-oxopyridine-3-carboxamide derivatives. To further study the SAR of 101, structural modification was conducted respectively by fluorine atom or by chlorine atom in *ortho* position of the benzylic group at N-1 position and by a cycloheptane-carboxamide at C-3 position of the central core, showed positive allosteric behavior on CB2.^87^
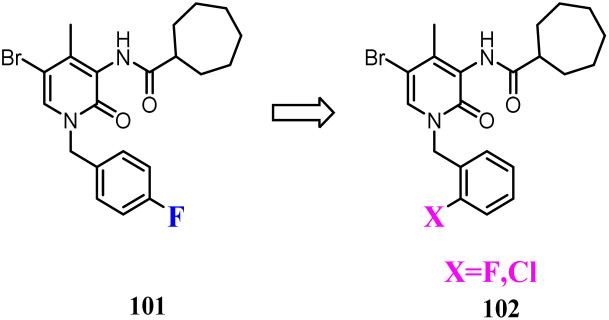


Compound 103 was found by ligand-based virtual screen, which was a moderate activity CB2 agonist with high selectivity. To obtain the greater CB2 potency with appropriate physicochemical and ADME properties for future evaluation *in vivo*, Russell' team replaced the alkyl chain with aryl chain.^88^ Several new examples exhibited high levels of activity (EC_50_ < 200 nM) and binding affinity (*K*_i_ < 200 nM) for CB2 without detectable activity at the CB1. The most promising compound 104 presented favorable metabolic stability *in vitro* and absorption properties along with a clean selectivity profile when evaluated against a panel of GPCRs and kinases.
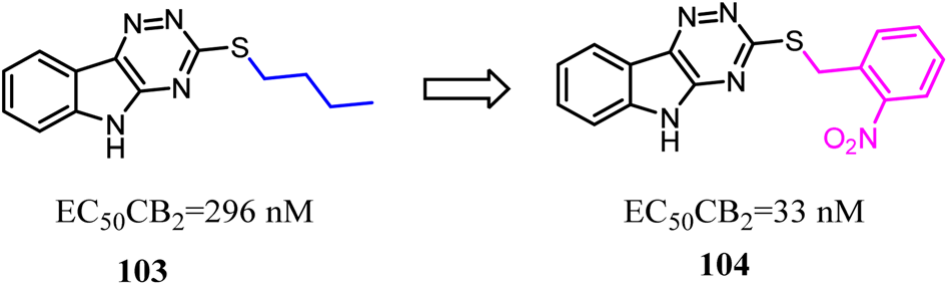


Compound 105 was discovered by using 3D pharmacophore database searches and was biologically confirmed as a new class of CB2 inverse agonist.^89^ Xie's team chosen compound 105 as the lead compound based on the SAR study. Subsequently, these derivatives were designed and synthesized through lead compound optimization by modifying the rings A–C and the SAR studies of dominant structure. They introduced the different alkyl groups into *para*-position of C ring and the directly improve the selectivity for CB2. Among them, compound 106 showed 72% inhibition activity even at the low concentration of 0.1 μM.
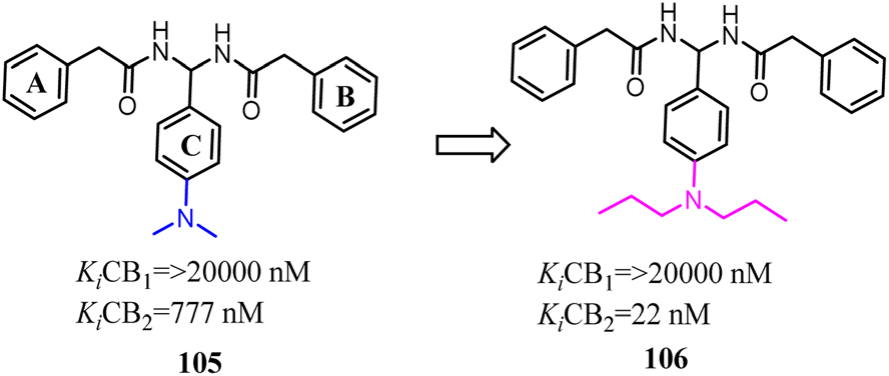


### 2.6 Agonist reversal antagonist

Relatively small structural modifications of CB2 ligands could lead to a major change in their functional profiles.^90^ In the 4-quinolone-3-carboxamide series of CB ligands, replacement of an isopropyl group at the 6-position with a furan-2-yl altered its CB2 functional profile from an agonist to an inverse agonist in GTPγS assay.^91–93^ Similarly, for the quinolone-2,4(1*H*,3*H*)-diones, C5- or C8-methyl substitutions were reported as CB2 agonists, while the C6- or C7-methyl substituted compounds acted as antagonists.^94^
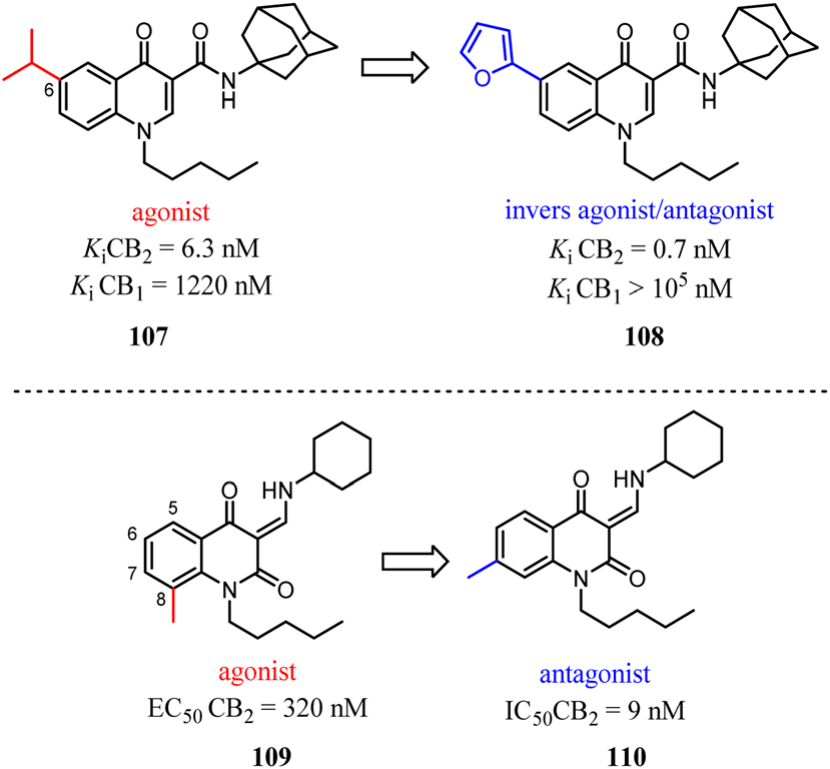


In our previous report, we found that a 7-methoxy or 7-methylthio substitution at the 3-amidoalkylindole 111 could form potent CB2 antagonists (112 or 113, IC_50_ = 16–28 nM) without observable agonist activity for CB2. We next performed flexible docking simulations to predict the receptor–ligand interactions between compound 111 or 112 and CB2 based on the homology model of CB2 active state,^95^ Our docking simulations also suggested that the agonist 111 would have a different interaction pattern with the antagonist 112 for their binding to the CB2 receptor.^58^
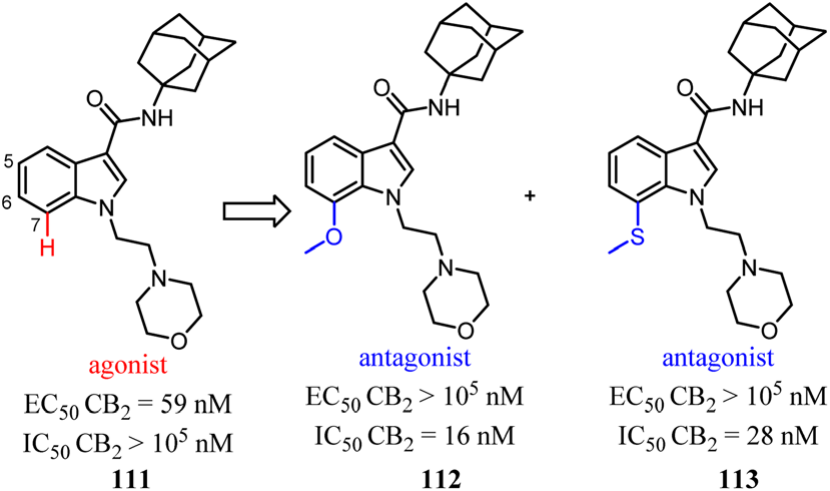


Corelli' team took the advantage of previous findings on structure activity/selectivity relationships for a class of 4-quinolone-3-carboxamides, and further structural modifications of the heterocyclic scaffold indicated when the 8-methy was replaced by 8-methoxy, the affinity and selectivity for CB2 were significantly improved, the derivative compound 115, evaluated *in vivo* in the formalin test, behaved as an inverse agonist by reducing at a dose of 6 mg kg^−1^ the second phase of the formalin-induced nocifensive response in mice.^96^
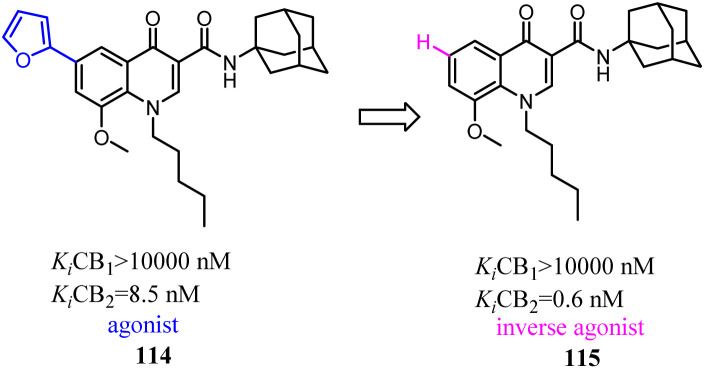


Vernall's team conducted the exploration of linker and fluorophore attachment, which belonged to alkyl indole-based CB2 tools. During the process of the project research, they found that shifted methoxy from C-5 to C-7 of indole core, the function would exchange from agonism to antagonism.^97^
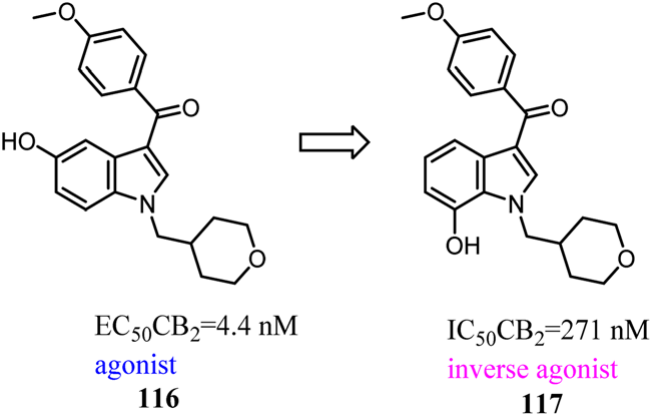


Baraldi's team identified the heteroaryl-4-oxopyridine/7-oxopyrimidine derivatives as highly potent and selective CB2 ligands, showing that the pharmacodynamics of the new compounds were controlled by the nature of the heterocycle core.^98^ They synthesized and evaluated the biological activity of 7-oxo-4-pentyl-4,7-dihydro-[1,2,4]triazolo[1,5-*a*]pyrimidine-6-carboxamide derivatives that led to the identification of novel CB2 inverse agonists. cAMP experiments on CB2 expressed in CHO cells revealed that introduction of structural modifications at position 2 of triazolopyrimidine template changes the functional activity from partial agonism to inverse agonism.
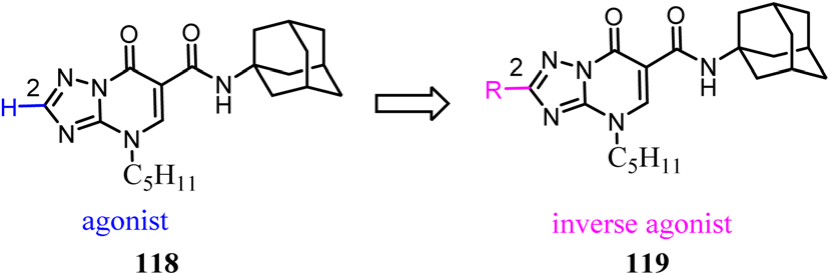


Manera's group identified 1,8-naphthyridin-2(1*H*)-one-3-carboxamide as a new scaffold which was very suitable for the development of new potent and selective CB2 ligands.^99^ They described a few additional derivatives in which the same central scaffold has been variously functionalized in position 6. All these novel ligands exhibited high selectivity and affinity in the nanomolar range for CB2. Furthermore, they found that the introduced substituents in C-6 position of the naphthyridine scaffold would determine a functionality switch from agonist to antagonists/inverse agonists. Finally, docking studies showed that the difference between the pharmacology of these ligands may be in the ability/inability to block the Toggle Switch W6.48(258) transition.
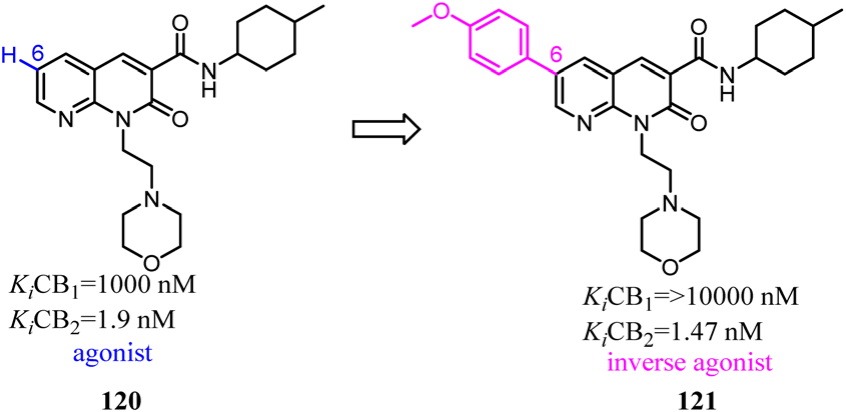


Based on a series of 4-oxo-1,4-dihydroquinoline-3-carboxamide served as selective CB2 agonists, Millet's group used the medicinal chemistry approach to develop a series of CB2 inverse agonists with a 4-oxo-1,4-dihydropyridine scaffold. The reported compounds exhibited high affinity and selectivity at the CB2. Furthermore, they concluded that the functionality of this series compounds was determined by its C-6 substituents because agonists bore a methyl or a *tert*-butyl group and inverse agonists, a phenyl or 4-chlorophenyl group, respectively. At last, *in silico* studies suggested that the C-6 substituent could modulate the conformation of W6.48 known to be critical in GPCR activation.^100^
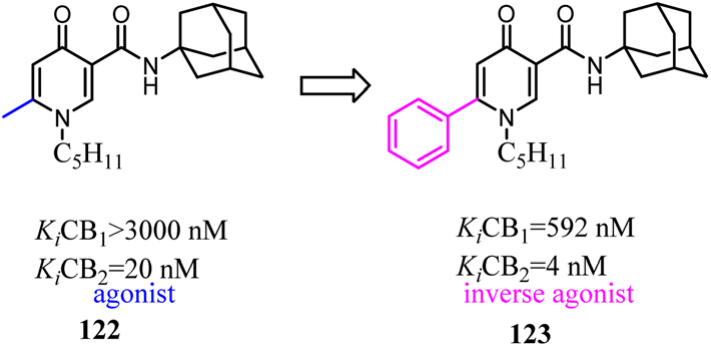


To further explore the SAR of biphenylic carboxamides which were reported by Bertini's team,^101^ they conducted the optimization of the biphenylic carboxamides. 18 Biphenylic carboxamides as novel CB2-selective ligands were synthesized, and their pharmacological profiles were also evaluated.^102^ The results indicated that the functional activity of these ligands was strongly influenced by the nature of the substituent at position 4′ and 5 in the biphenyl scaffold. This study provided a novel complete toolbox of CB2 functional modulators that derive from the same chemical scaffold. Position 5 seemed to be responsible for the agonist or inverse agonist behaviour independently of the substituent in position 4′, with the exception of the methoxyl group which transformed both full agonists and inverse agonists into neutral antagonists. This study provided a novel complete toolbox of CB2 functional modulators that derive from the same chemical scaffold.
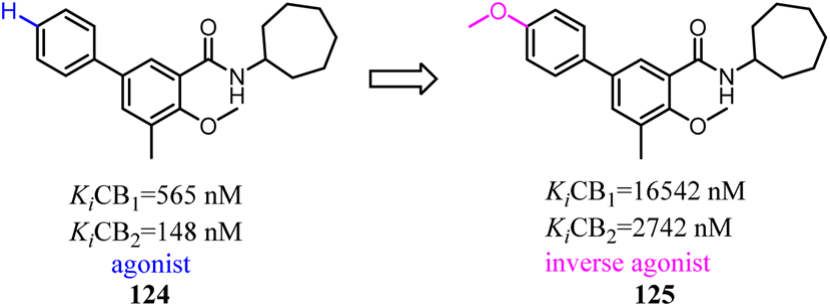


Brière's team has established a synthetic strategy, which achieved the scaffold hopping approach, and was used for construction of C4-benzyl pyrazolines and derivatives from readily available hydrazones and enones.^103^ The obtained family of pyrazolines featured a significant CB2/CB1 selectivity in favor of CB2 receptors. This was closely related to pyrazole SR144528 inverse agonist/antagonist. Furthermore, this hCB2 selectivity was unique within the pyrazoline CB ligands although the affinity ranging from 251 to 689 nM remained to be improved.
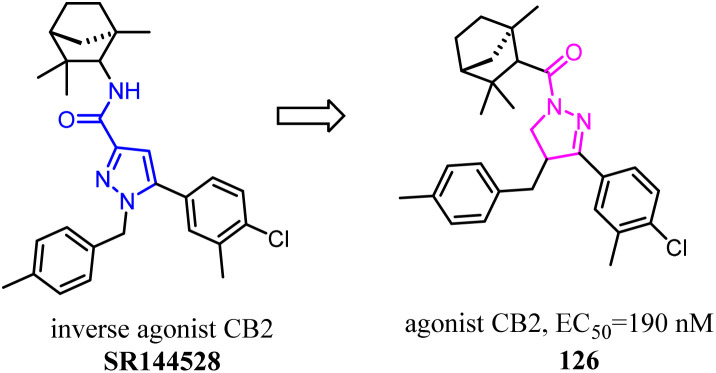


Compound 127 exhibited high affinities for both CBs with agonism for CB2. To improve the selectivity for CB2, Dudley's team shifted the 3-formyl indole from C4 position of indene to C5 position. As the goal was achieved, another surprise was discovered that the agonist turned into an antagonist. Considering that some indole CB2 agonists were classified as abuse drug and CB2 antagonists were promising but rare, hence, functionality switch would be a promising strategy to discover more CB2 antagonists.^104^
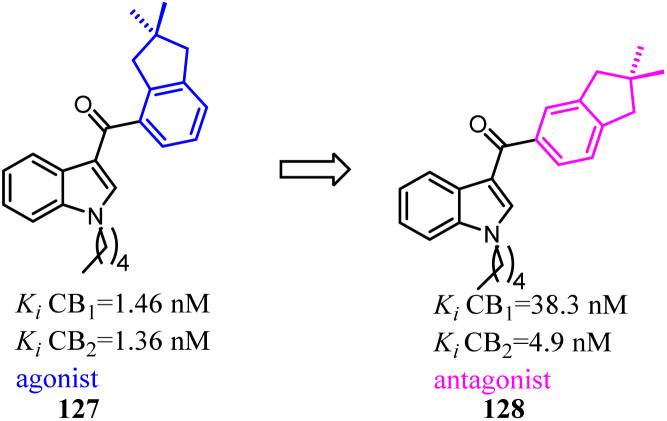


## 3 Conclusion and outlook

At present, most natural and endogenous CB2 agonists are non-selective, while most selective CB2 agonists are synthesized based on non-selective CB2 agonists through structural modification and optimization. In this process, several equilibria must be struck, such as selectivity and lipophilic/hydrophilic, activity and hydrophilic/lipophilic, and lipophilic and hydrophilic. One function of the various examples listed above is to find this balance. Structurally, the agonist binding pockets of CB1 and CB2 are very similar, including binding modes and key amino acids, which may explain the poor selectivity of cannabinoid receptor agonists structurally. Although high selectivity and high activity CB2 receptor agonists can be obtained by using traditional structural optimization methods, the binding efficiency of ligand and target and the structural diversity are generally relatively low, which greatly increases the cost of preclinical research. In our opinion, *de novo* drug design, combining with deep learning, allows precise design of selective CB2 receptor agonists, which could reduce the unnecessary structural optimizations and essentially lower the cost of drug development. Krishnan *et al.* proposed a deep learning-based approach to *de novo* drug design, in which knowledge of the structure of the active site of the target protein is sufficient for new molecular design.^105^ This method was widely used to generate entirely new molecules for any protein whose structure was known. In the near future, we predict that the design of selective CB2 receptor agonists from *de novo* drug design by artificial intelligence may lead to the introduction of more ligands into the clinic and market. So far, no selective CB2 receptor ligand has been approved to market as a drug. Therefore, the research direction can be adjusted to FAAH inhibitors, peripherally-restricted CB1 antagonists, neutral CB1 antagonists, allosteric modulators, *etc.*

## Conflicts of interest

The authors declare that they have no known competing financial interests or personal relationships that could have appeared to influence the work reported in this paper.

## Abbreviations

QSAR: Quantitative structure–activity relationship
CoMFA: Comparative molecular field analysis
ADMET: Absorption, distribution, metabolism, excretion, toxicity
AMP: Cyclic adenosine monophosphate
DSS: Dextran sulfate sodium
GTPgS: Guanosine 50-*O*-[gamma-thio]triphosphate
MCP-2: Monocyte chemotactic protein-2
LPS: Lipopolysaccharide
SAR: Quantitative structure–activity relationship
FAAH: Fatty acid amide hydrolase
TRPV1: Vanilloid receptors
TRPA1: Transient receptor potential cation channel
Phe: Phenylalanine
Trp: Tryptophan
Gly: Glycine acid
HL: Human promyeloid leukemia
CNS: Central nervous system
*K*_i_
: Dissociation constant
PET: Polyethylene terephthalate
GPCR: G protein-coupled receptors

## Supplementary Material
